# Characterization of GABAergic Neurons in the Mouse Lateral Septum: A Double Fluorescence In Situ Hybridization and Immunohistochemical Study Using Tyramide Signal Amplification

**DOI:** 10.1371/journal.pone.0073750

**Published:** 2013-08-13

**Authors:** Changjiu Zhao, Brian Eisinger, Stephen C. Gammie

**Affiliations:** 1 Department of Zoology, University of Wisconsin-Madison, Madison, Wisconsin, United States of America; 2 Neuroscience Training Program, University of Wisconsin-Madison, Madison, Wisconsin, United States of America; University of Minnesota, United States of America

## Abstract

Gamma-aminobutyric acid (GABA) neurotransmission in the lateral septum (LS) is implicated in modulating various behavioral processes, including emotional reactivity and maternal behavior. However, identifying the phenotype of GABAergic neurons in the CNS has been hampered by the longstanding inability to reliably detect somal immunoreactivity for GABA or glutamic acid decarboxylase (GAD), the enzyme that produces GABA. In this study, we designed unique probes for both GAD65 (GAD2) and GAD67 (GAD1), and used fluorescence in Situ hybridization (FISH) with tyramide signal amplification (TSA) to achieve unequivocal detection of cell bodies of GABAergic neurons by GAD mRNAs. We quantitatively characterized the expression and chemical phenotype of GABAergic neurons across each subdivision of LS and in cingulate cortex (Cg) and medial preoptic area (MPOA) in female mice. Across LS, almost all GAD65 mRNA-expressing neurons were found to contain GAD67 mRNA (approximately 95-98%), while a small proportion of GAD67 mRNA-containing neurons did not express GAD65 mRNA (5-14%). Using the neuronal marker NeuN, almost every neuron in LS (> 90%) was also found to be GABA-positive. Interneuron markers using calcium-binding proteins showed that LS GABAergic neurons displayed immunoreactivity for calbindin (CB) or calretinin (CR), but not parvalbumin (PV); almost all CB- or CR-immunoreactive neurons (98-100%) were GABAergic. The proportion of GABAergic neurons immunoreactive for CB or CR varied depending on the subdivisions examined, with the highest percentage of colocalization in the caudal intermediate LS (LSI) (approximately 58% for CB and 35% for CR). These findings suggest that the vast majority of GABAergic neurons within the LS have the potential for synthesizing GABA via the dual enzyme systems GAD65 and GAD67, and each subtype of GABAergic neurons identified by distinct calcium-binding proteins may exert unique roles in the physiological function and neuronal circuitry of the LS.

## Introduction

A thorough understanding of the phenotype of neurons in a given brain region is important in clarifying structure, neuronal connectivity, and functional significance. Gamma-aminobutyric acid (GABA) ergic neurons are ubiquitously present throughout the mammalian brain, and GABA has been broadly involved in the modulation of multiple behaviors, including maternal behavior, anxiety, depression, aggression, and stress response [1–9]. However, reliable and consistent detection of the cell bodies of GABAergic neurons in certain brain regions has long been difficult to achieve by immunohistochemical assays with antisera against GABA or glutamic acid decarboxylase (GAD), the enzyme for GABA synthesis and a specific marker for GABAergic neurons [10–12]. Therefore, precise mapping of the distribution and identification of phenotype of GABAergic neurons in the CNS have been hindered by technical difficulties in reliably identifying somal GABA immunoreactivity using antibody-based detection methods. Colocalization study of GAD65/GAD67 or GABA with other markers for specific neuronal cell types was further complicated by the diffusely distributed punctate structures that correspond to axon terminals, and few cell bodies are immunoreactive for GAD65/GAD67 [13–16] or GABA [17].

Many improved immunohistochemical approaches have been widely employed to enhance the sensitivity of detection for GABAergic neurons, including the use of fixative containing relatively high concentration of glutaraldehyde (0.5-2.5%) to preserve GABA immunoreactivity by binding GABA rapidly to tissue [18–22], colchicine treatment, which blocks axonal transport of GABA/GAD and thus greatly enhances somatic labeling in GABA-immunoreactive neurons [11,23–26], and the exclusion of detergents like Triton X-100 from the immunohistochemical procedures to increase the staining intensity of cell bodies [11,21,22,27,28]. Although the above improved methods have been developed, the low level of cellular resolution of positively labeled cell bodies is still not sufficient to allow for a precise determination. More recently, an animal model of GAD67-green fluorescent protein (GFP) knock-in mice was generated to identify GABAergic neurons [29–31]. Although using transgenically expressed phenotypic markers to label cell types has been demonstrated to be a highly effective tool, in situ hybridization (ISH) techniques appear to have an advantage in faithfully reflecting native gene expression over transgenically controlled reporters, as not all the GABAergic cells expressed transgenic protein markers [31–34].

In comparison with immunohistochemical methods, ISH method is more sensitive in labeling small GABAergic neurons and neuronal populations that express low levels of GAD mRNAs [35]. More importantly, earlier nonradioactive ISH studies using riboprobes specific for each of the two GAD mRNAs allows detection of the cell bodies of the vast majority of GABAergic neurons, thus making accurate quantification of GABAergic neurons possible [35,36]. Fluorescence ISH (FISH) with tyramide signal amplification (TSA) has been established to be a useful means of detecting low abundance target mRNAs and colocalization of overlapping genes in a single cell based on its dramatically enhanced sensitivity of detection and high cellular resolution [37–41].

In the present study, we aimed to characterize the localization, morphology, expression level and chemical phenotype of GABAergic neurons in the lateral septum (LS) more precisely in mice. To this end, we employed double FISH and immunohistochemistry (IHC) with TSA. We chose LS as a target neural site in that LS is a core brain region enriched with GABA as well as a variety of neurotransmitters, neuropeptides and receptors [42,43] and has been linked to multiple roles in regulating various behaviors [9,42,44–46]. We first assessed the distribution of LS GABAergic neurons identified by mRNAs for GAD65 and GAD67 and the colocalization of the two isoforms of mRNAs in female mice. In addition, we determined the percentage of GABA-positive cells using the neuronal marker NeuN. Finally, we examined the degree of colocalization of GABA with calcium-binding proteins calbindin (CB), calretinin (CR) and parvalbumin (PV) (markers for GABAergic interneurons) in the LS neurons. We also examined cingulate cortex (Cg) and medial preoptic area (MPOA) from the same tissue slices used for LS analysis, as this aids in expanding results from our approaches to additional regions.

## Results

### Distribution and staining pattern of GAD65 and GAD67 mRNA-expressing neurons in the LS, Cg, and MPOA

**Figure 1:**
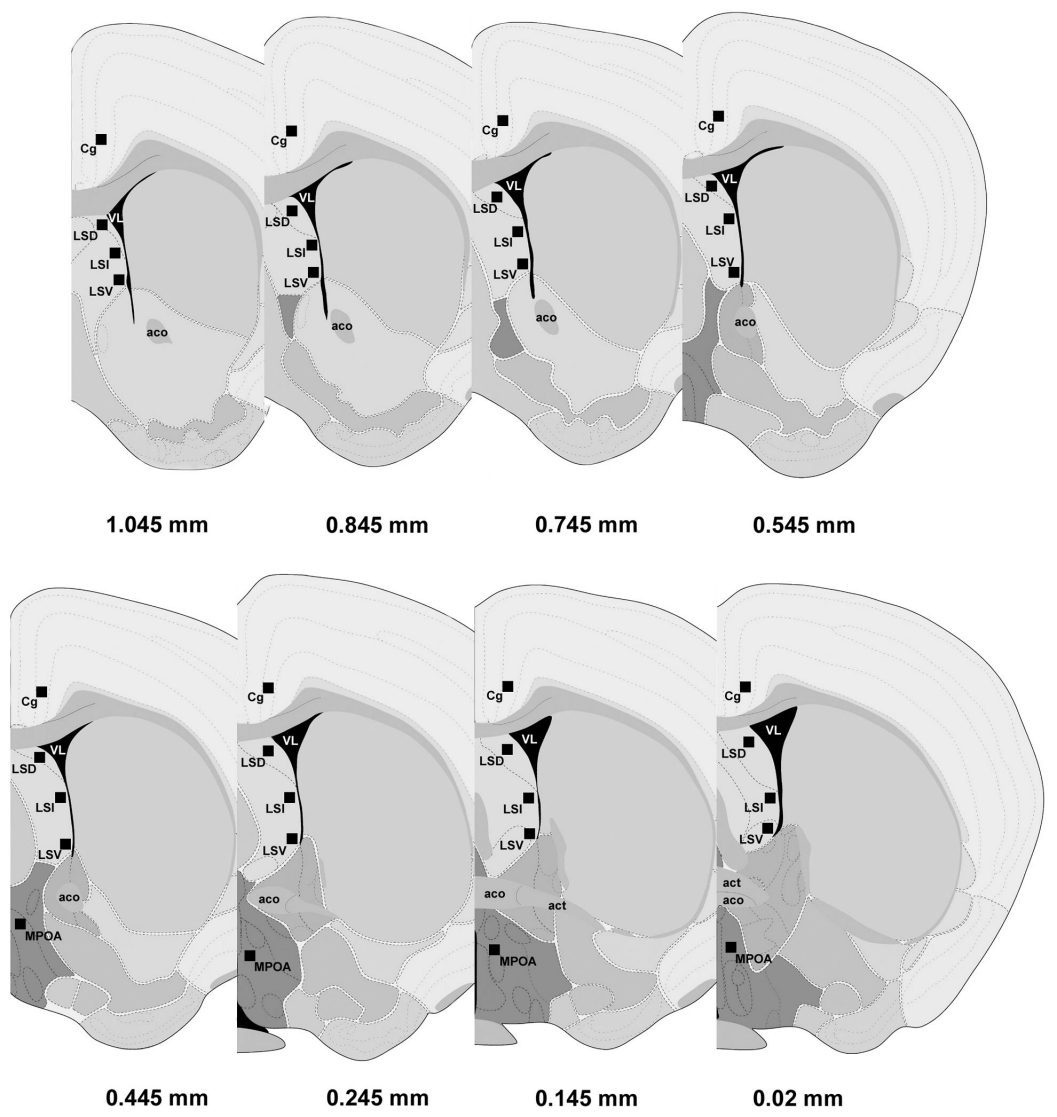
Schematic representation of the brain areas (black boxed regions) in which expression of mRNA and protein immunoreactivity as well as colocalization was examined. Figures are adapted and modified from The Allen Mouse Brain Atlas (Reference Atlas Version 1, 2008). Nomenclature is obtained from Paxinos and Franklin [108]. Distance from bregma in the rostrocaudal planes is indicated. Abbreviations: aco, anterior commissure, olfactory limb; act, anterior commissure, temporal limb; Cg, cingulate cortex; LSD, dorsal part of lateral septal nucleus; LSI, intermediate part of lateral septal nucleus; LSV, ventral part of lateral septal nucleus; MPOA, medial preoptic area; VL, lateral ventricle.

**Figure 2:**
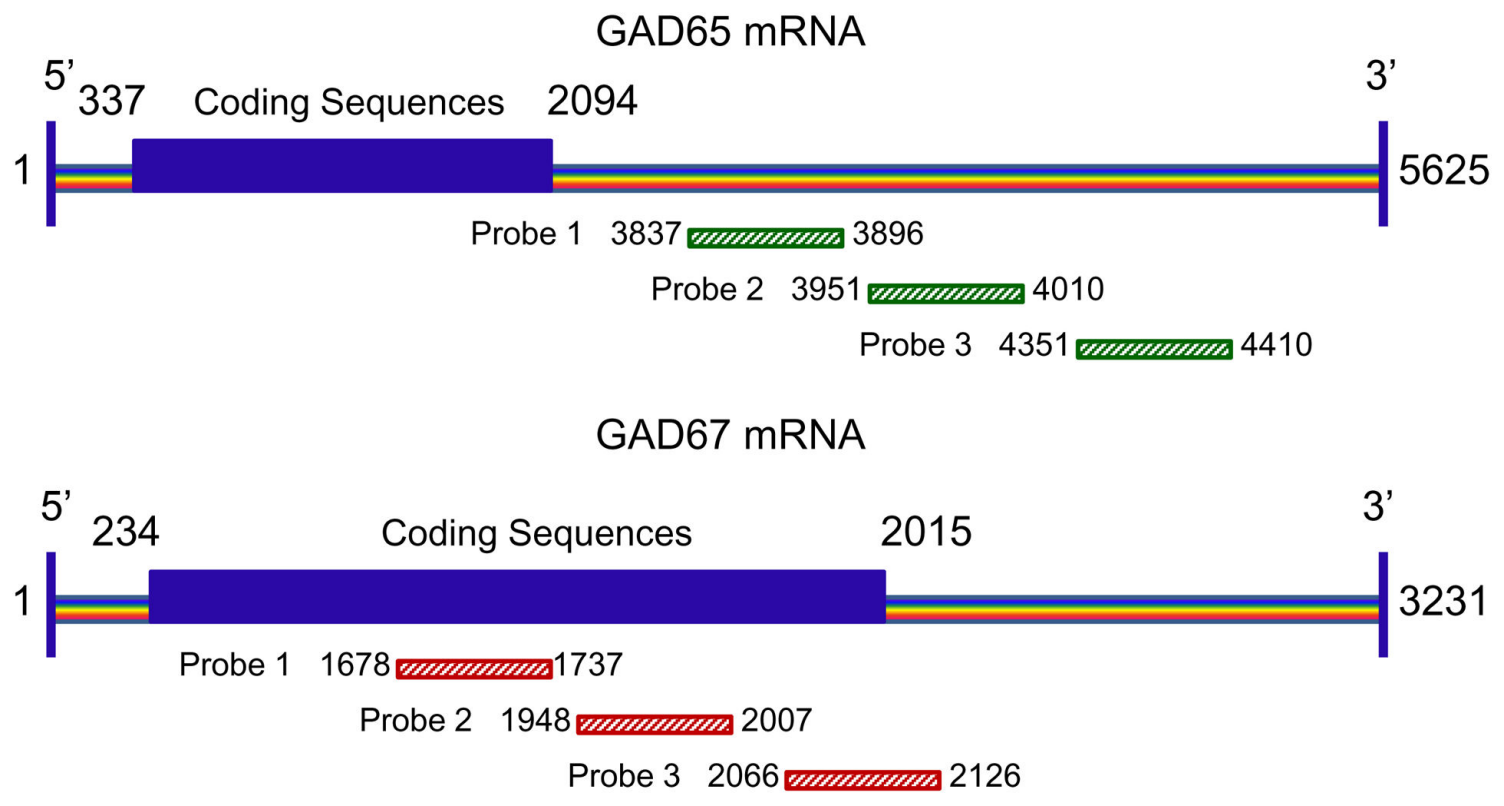
Schematic diagram depicting probe design for GAD65 and GAD67 in situ hybridization assay. For each gene, three sets of oligonucleotide probes were generated to target three distinct sequences of each mRNA. Recognition of GAD65 and GAD67 mRNA sequences and length covered by each oligonucleotide probe are indicated. There was no overlap of sequences among all the probes and the probes designed for the two GADS had no homology to one another to ensure the specificity of the probe. The GAD67 probes were labeled with digoxigenin, while GAD65 probes were labeled with either biotin or digoxigenin. Note that probes for GAD65 target the mRNA 3’-non-coding sequences, whereas probes for GAD67 primarily recognize the mRNA 3’-coding sequences (blue box).

**Figure 3:**
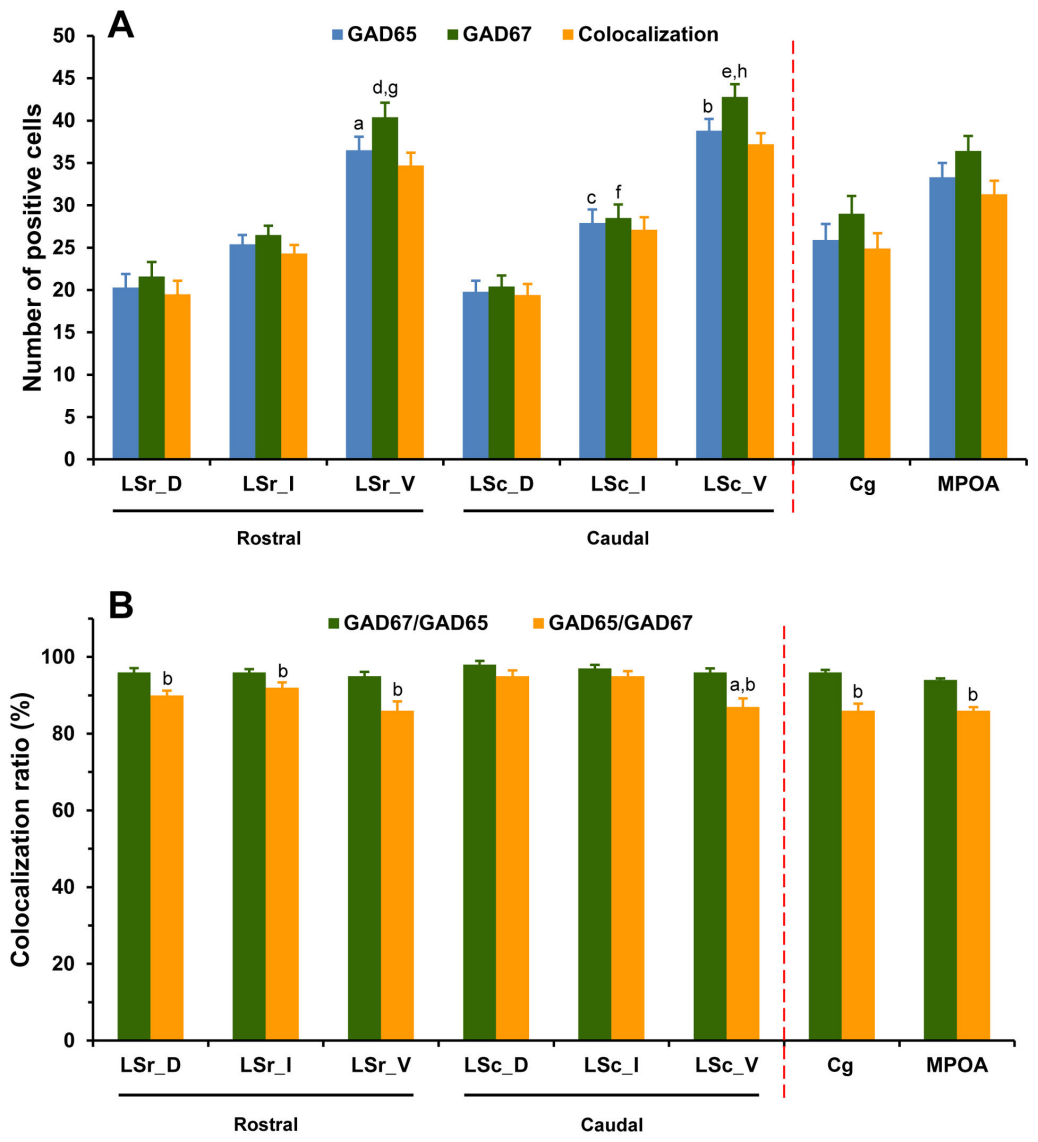
Expression and colocalization of neurons expressing mRNAs for GAD65 and GAD67 in the LS, Cg and MPOA. The number (A) and colocalization ratio (B) is presented. Note that mRNAs for both GAD65 and GAD67 are colocalized in the vast majority of neurons of the three brain regions. Each bar represents the mean+SEM obtained from six mice. Colocalization ratio of GAD67/GAD65 was calculated by dividing the number of neurons expressing both GAD65 and GAD67 mRNAs (colocalization) by total number of neurons expressing GAD65 mRNA, and multiplying by 100. A: ^a^ *p* < 0.001 *versus* LSr_D and LSr_I, ^b^ *p* < 0.05 *versus* LSc_D and LSc_I, ^c^ *p* < 0.001 *versus* LSc_D, ^d^ *p* < 0.001 *versus* LSr_D and LSr_I, ^e^ *p* < 0.05 *versus* LSc_D and LSc_I, ^f^ *p* < 0.001 *versus* LSc_D, ^g^ *p* < 0.001 *versus* GAD65 in LSr_V, ^h^ *p* < 0.001 *versus* GAD65 in LSc_V; B: ^a^ *p* < 0.05 *versus* LSc_D and LSc_I, ^b^ *p* < 0.05 *versus* GAD67/GAD65.

**Figure 4:**
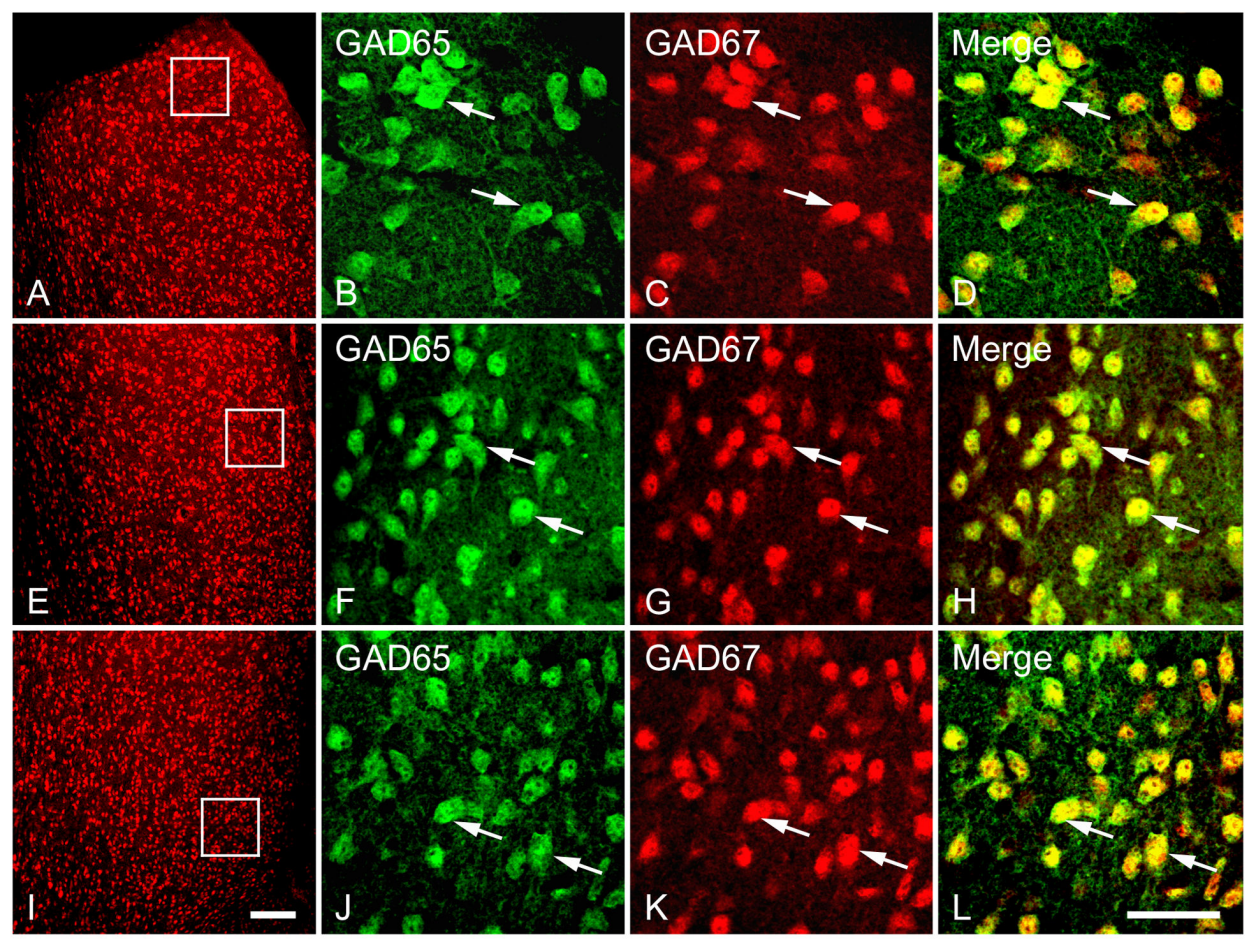
Double fluorescence in situ hybridization labeling of GAD65 and GAD67 mRNA-expressing neurons in the LSD (A–D), LSI (E–H) and LSV (I–L). Low magnification images of the white boxed regions (A, E and I) show neurons expressing mRNAs for GAD65 and GAD67 were counted for colocalization analysis. High magnification images show the colocalization of neurons containing both GAD65 and GAD67 mRNAs in the LSD (B–D), LSI (F–H) and LSV (J–L). Two typical examples of neurons coexpressing mRNAs for GAD65 and GAD67 in each subdivision are indicated in arrows. Note that in all three subdivisions of the LS, GAD65 and GAD67 mRNAs are highly colocalized in a single neuron. Scale bars = 150 µm in A, E and I; 50 µm in B–D, F–H, J-L.

**Figure 5:**
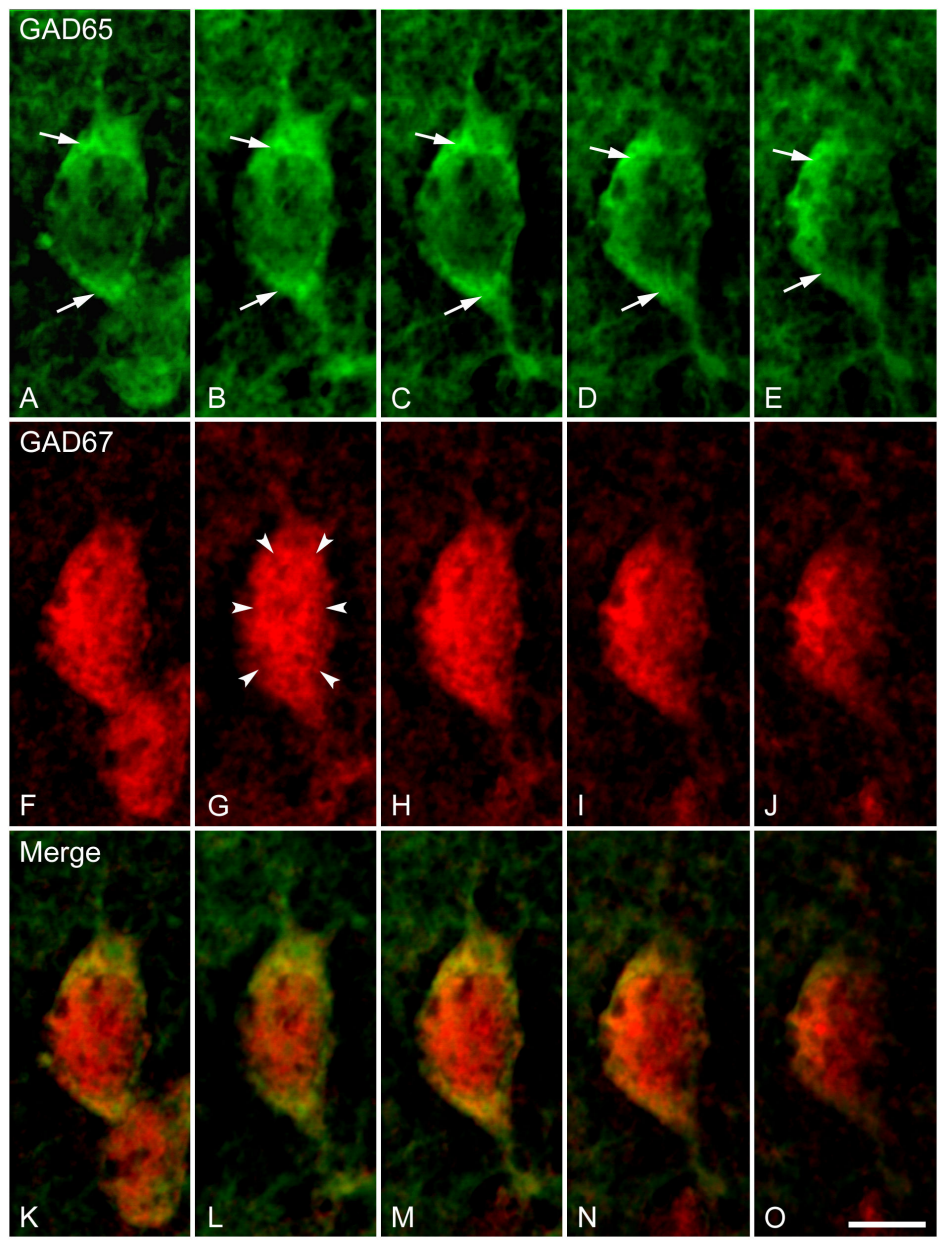
High magnification photomicrographs of z-series stacks displaying double fluorescence in situ hybridization labeling of GAD65 and GAD67 mRNA-expressing neurons in a single neuron of the LSI. Images represent a series of four sequential photomicrographs (from B-E, G-J and L-O) that were captured at a distance of 0.5 micron apart through the entire thickness of the brain section, while images in A, F and K were projected images from four sequential photomicrographs correspondingly. Arrows indicate intensely labeled GAD65 mRNA at the periphery of cell bodies and axon initial segment, and arrowheads indicate robust and even labeling throughout the soma of the GAD67 mRNA-expressing cells. Scale bar = 250 µm.

**Figure 6:**
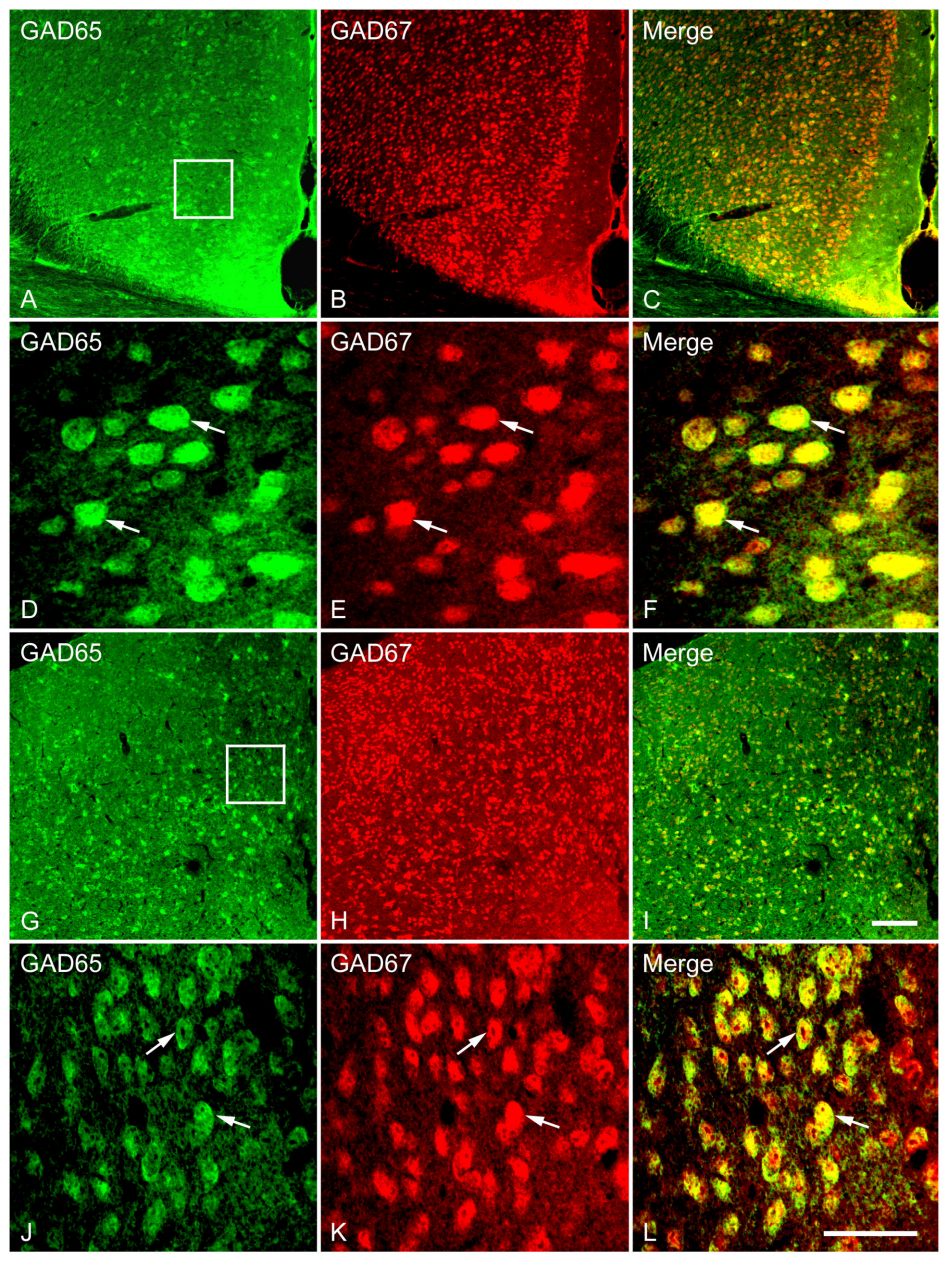
Double fluorescence in situ hybridization labeling of GAD65 and GAD67 mRNA-expressing neurons in the Cg (A–F) and MPOA (G–L). Low magnification images of the white boxed regions (A–C, G–I) show the GAD65 and GAD67 mRNA-expressing neurons were counted for colocalization analysis. High magnification images show the colocalization of GAD65 and GAD67 mRNA-expressing neurons in the Cg (D–F) and MPOA (J–L). Two typical examples of double-labeled neurons, indicating the colocalization of GAD65 and GAD67 mRNA in the Cg and MPOA are indicated in arrows. Note that mRNAs for GAD65 and GAD67 are highly coexpressed in a single cell. Scale bars = 150 µm in A-C, G-I; 50 µm in D–F, J-L.

In this study, we focused on multiple subdivisions of LS while also evaluating Cg and MPOA (Figure 1). We designed oligonucleotide probes targeted either to GAD65 mRNA or GAD67 mRNA with high specificity, such that no possible overlap in recognition occurred (Figure 2). Neurons containing mRNAs for GAD65 and GAD67 were highly expressed throughout the entire rostrocaudal extent of three subdivisions [e.g., dorsal part of the lateral septum (LSD), intermediate part of the lateral septum (LSI), and ventral part of the lateral septum (LSV)]. One-way ANOVA revealed a significant difference in the density of GAD65 [*F*(7,40) = 21.79, *p* < 0.001] and GAD67 [*F*(7,40) = 25.68, *p* < 0.001] mRNA-expressing neurons among subdivisions of the LS, Cg and MPOA. As shown in Figure 3A, at both the rostral and caudal levels of LS, neurons labeled for either type of mRNA were more numerous in the LSV than in the LSD and LSI, as well as in the caudal LSI relative to caudal LSD. In general, within the LS, neurons containing GAD67-mRNA appeared to slightly outnumber those expressing GAD65-mRNA. Examples of a significantly higher number of GAD67 neurons versus GAD65 cells in the rostral and caudal LSV were observed. The morphology, size and orientation pattern of neurons expressing mRNAs for GAD65 and GAD67 were quite similar. They were small- and medium-sized round or oval cells, with intensely labeled cell bodies (both cytoplasm and nucleus) and/or lightly labeled proximal dendritic processes. In the LSD (Figure 4B-C) and LSI (Figure 4F-G), oval cells frequently displaying one or two dendrites that stemmed from the somata predominated, while the overwhelming majority of round cells were detected in the LSV (Figure 4J-K). However, no appreciable difference in the relative intensity of labeling for the two GAD mRNAs was detected between the rostral and caudal levels in the three subdivisions of LS. A lack of punctate structures corresponding to presumptive axon terminals was also evident. Although neurons labeled for GAD65 mRNA were not readily distinguished from cells labeled for GAD67 mRNA based on their profound similarity in morphological features, there were still a few distinct characteristics between the two cell types. One striking difference is that more intense labeling on GAD65 mRNA-expressing neurons was typically detected at the periphery of cell bodies and axon initial segment, whereas robust labeling was more evenly distributed throughout the soma of the GAD67-labeled cells, with occasional existence in the proximal dendritic processes, as illustrated in a typical example of a neuron obtained from z-series stacks (Figure 5). Of interest, the staining pattern of GAD67 mRNA coincides with its immunoreactivity [47]. Neurons expressing mRNAs for GAD65 and GAD67 were found to be abundantly expressed in the Cg (Figure 6A–F) and MPOA (Figure 6G–L), and shared similar distribution and staining pattern with the LS.

### Colocalization of GAD65 and GAD67 mRNA-expressing neurons in the LS, Cg and MPOA

To estimate the percentage of colocalization of GAD65 and GAD67 mRNAs in the same cells, we employed double FISH experiments labeling for these two cell types. As can be seen in Figure 3B, GAD65 and GAD67 mRNAs were coexpressed in the vast majority of neurons within the entire LS, Cg, and MPOA. Almost all GAD65-labeled neurons were also labeled for GAD67 (95-98%), whereas a fraction of GAD67-positive cells did not contain GAD65 (5-14%). When comparing the colocalization ratio of GAD67/GAD65 and GAD65/GAD67 among subdivisions of the LS, Cg and MPOA at a given rostrocaudal level, a significant difference in GAD65/GAD67 [*F*(7,40) = 5.82, *p* < 0.001], but not in GAD67/GAD65 [*F*(7,40) = 1.55, *p* = 0.18] was observed. In the caudal LS, GAD67-labeled neurons that were also positively labeled for GAD65 were less numerous in the LSV than in LSD (*p* = 0.016) and LSI (*p* = 0.029). In general, the percentage of colocalization for GAD67/GAD65 tends to be higher than GAD65/GAD67 in all the brain regions of interest. One-way ANOVA revealed a higher ratio of colocalization of GAD67/GAD65 than GAD65/GAD67 in rostral LSD (*p* = 0.004), LSI (*p* = 0.039) and LSV (*p* = 0.006), caudal LSV (*p* = 0.003), Cg and MPOA (both *ps* < 0.001).

### Distribution, staining pattern, and co-localization of cell populations expressing GAD65/GAD67 (GAD) mRNAs and NeuN-immunoreactivity in the LS, Cg, and MPOA

**Figure 7:**
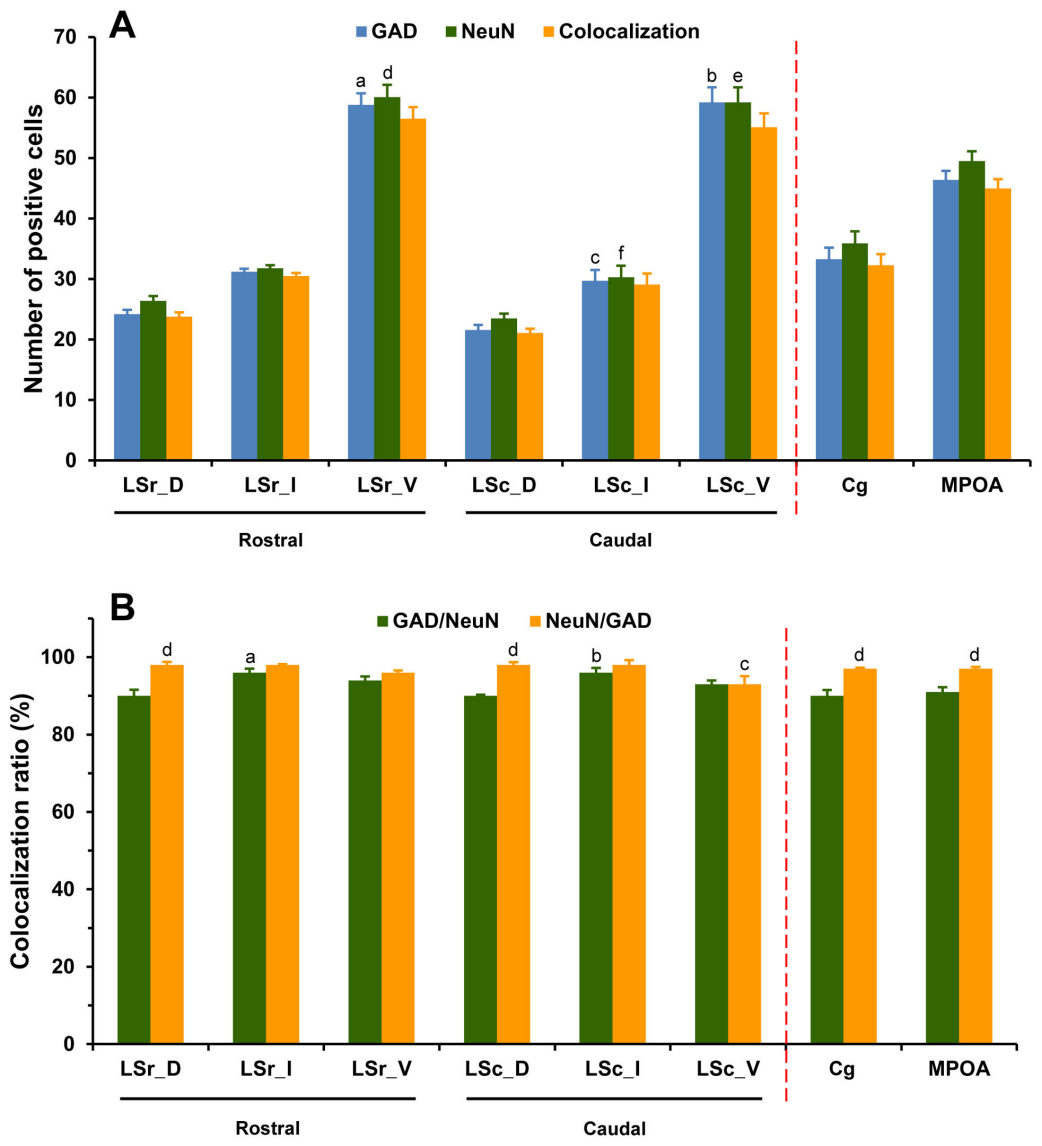
Expression and colocalization of cells expressing mRNAs for GAD65/GAD67 (GAD) and NeuN-immunoreactivity in the LS, Cg and MPOA. The number (A) and colocalization ratio (B) was calculated. Note that the vast majority of cells coexpressed GAD and NeuN within the three brain regions. Each bar represents the mean+SEM obtained from six mice. A: ^a^ *p* < 0.001 *versus* LSr_D and LSr_I, ^b^ *p* < 0.05 *versus* LSc_D and LSc_I, ^c^ *p* < 0.001 *versus* LSc_D, ^d^ *p* < 0.001 *versus* LSr_D and LSr_I, ^e^ *p* < 0.05 *versus* LSc_D and LSc_I, ^f^ *p* < 0.001 *versus* LSc_D; B: ^a^ *p* < 0.05 *versus* LSr_D, ^b^ *p* < 0.05 *versus* LSc_D, ^c^ *p* < 0.01 *versus* LSc_D and LSc_I, ^d^ *p* < 0.05 *versus* GAD/NeuN.

**Figure 8:**
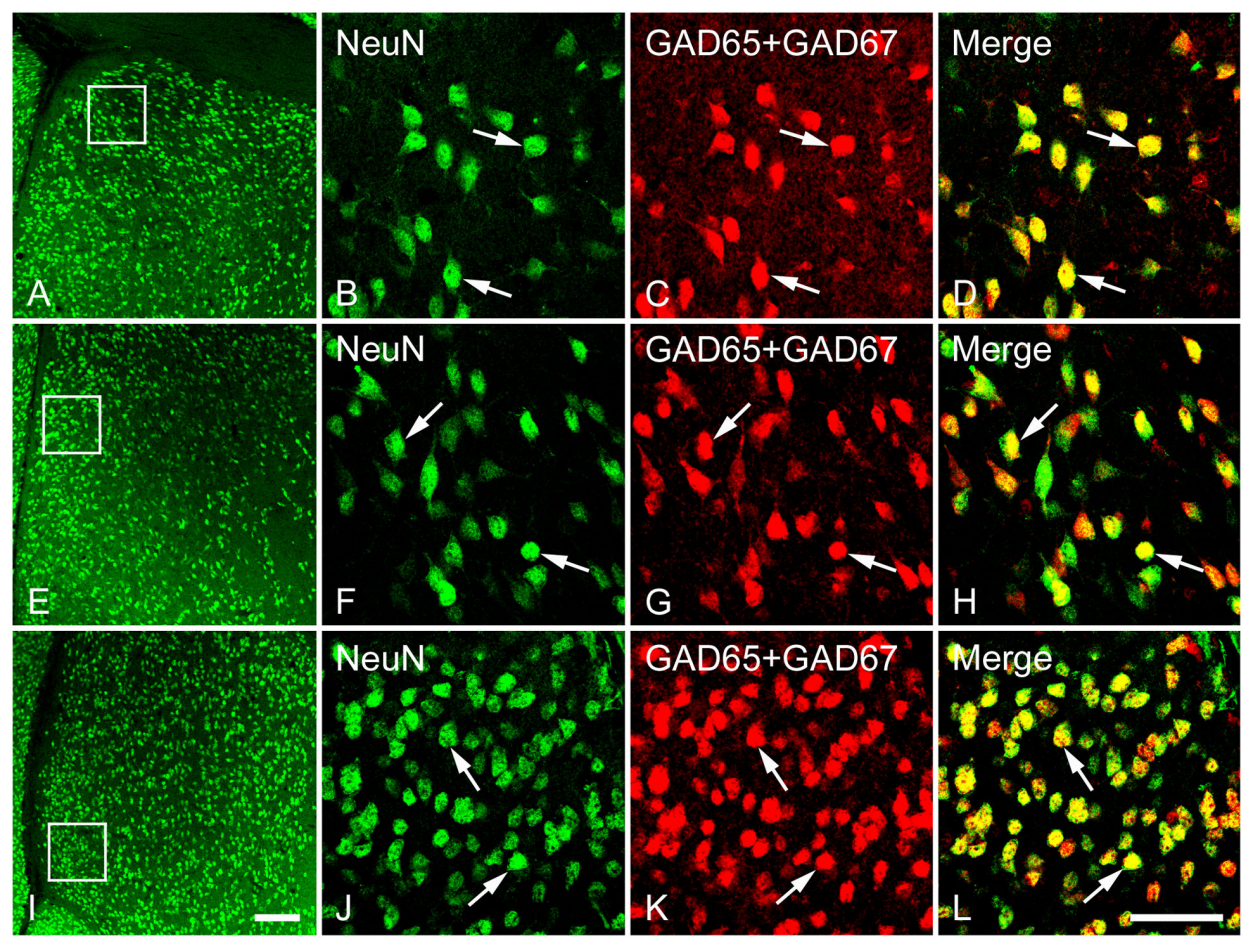
Double fluorescence in situ hybridization and immunohistochemistry labeling of neurons expressing mRNAs for GAD65/GAD67 (GAD) and NeuN-immunoreactivity in the LSD (A–D), LSI (E–H) and LSV (I–L). Low magnification images of the white boxed regions (A, E and I) show the GAD- and NeuN-expressing neurons were counted for colocalization analysis. High magnification images show the colocalization of GAD and NeuN in the LSD (B–D), LSI (F–H) and LSV (J–L). Two typical examples of double-labeled neurons, indicating the colocalization of GAD and NeuN in each subdivision are indicated in arrows. Note that in all three subdivisions of the lateral septum, GAD and NeuN are highly coexpressed in a single cell. Scale bars = 150 µm in A, E and I; 50 µm in B–D, F–H, J-L.

**Figure 9:**
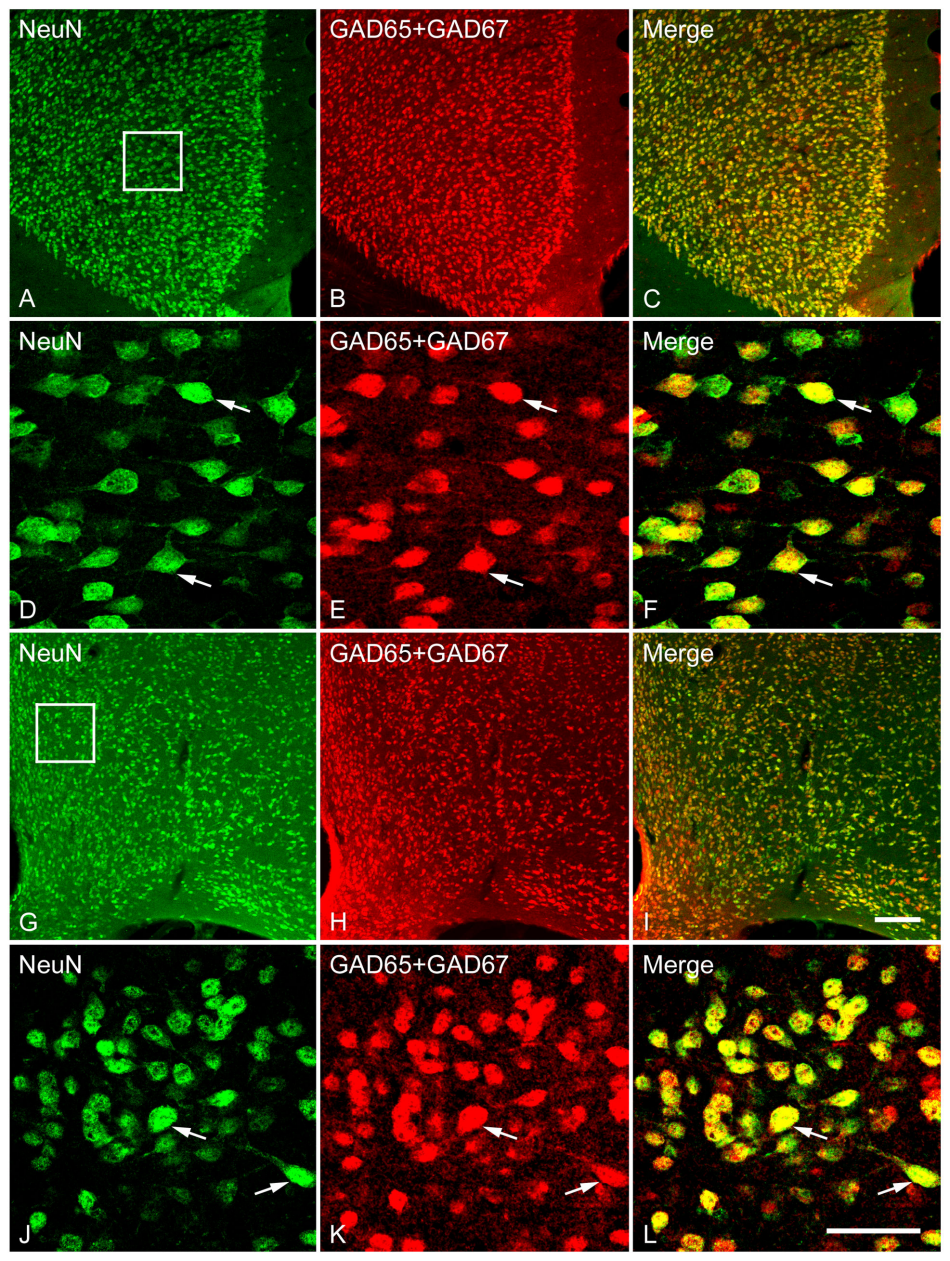
Double fluorescence in situ hybridization and immunohistochemistry labeling of neurons expressing mRNAs for GAD65/GAD67 (GAD) and NeuN-immunoreactivity in the Cg (A–F) and MPOA (G–L). Low magnification images of the white boxed regions (A–C, G–I) show the GAD- and NeuN-expressing neurons were counted for colocalization analysis. High magnification images show the colocalization of GAD- and NeuN-expressing neurons in the Cg (D–F) and MPOA (J–L). Two typical examples of double-labeled neurons in the Cg and MPOA are indicated in arrows. Note that GAD and NeuN are highly coexpressed in a single cell. Scale bars = 150 µm in A-C, G-I; 50 µm in D–F, J-L.

In order to examine the percentage of neurons in LS that contain GABA, we used antibodies that recognize the neuronal marker NeuN. For the double-labeling we used probes for both GAD65 and GAD67 as described above, but linked both to the common digoxigenin (DIG)-labeled probe to provide a unified signal for GABA cells using in situ hybridization approaches. The distribution and staining pattern of GAD mRNA-expressing cells was identical to those described above. NeuN-immunoreactive cells exhibited a similar distribution and staining pattern to cells expressing GAD mRNAs throughout the entire rostrocaudal extent of the LS. One-way ANOVA analysis revealed a significant difference in the density of cells labeled for GAD [*F*(7,40) = 87.05, *p* < 0.001] as well as NeuN [*F*(7,40) = 78.54, *p* < 0.001] among subdivisions of the LS, Cg and MPOA. As shown in Figure 7A, neurons labeled for either GAD or NeuN were more numerous in the LSV than in the LSD and LSI at both the rostral and caudal levels. The number of cells labeled for GAD or NeuN in caudal LSI was higher than in caudal LSD. However, the number of cells labeled for GAD or NeuN did not differ among three subdivisions between the rostral and caudal levels. In general, within the LS, no significant difference was found between the number of GAD-labeled cells and NeuN-immunoreactive ones, although NeuN-positive cells appeared to slightly outnumber GAD cells. The morphology, size and orientation pattern of NeuN-immunoreactive cells in the LS were quite similar to those expressing GAD mRNAs (Figure 8B-C, F-G, J-K), as mentioned above. Like LS, NeuN-immunoreactive neurons were found to be richly expressed in the Cg (Figure 9A–F) and MPOA (Figure 9G–L), and shared similar distribution and staining pattern with the LS.

Double labeling study revealed that more than 90% of neurons (determined via NeuN) in the LS were also GABA positive. As depicted in Figure 7B, almost all GAD mRNA-expressing cells also exhibited NeuN-immunoreactivity, indicating that these GABAergic cells are neurons. One-way ANOVA confirmed a significant difference in colocalization ratio of GAD/NeuN [*F*(7,40) = 4.93, *p* < 0.001] and NeuN/GAD [*F*(7,40) = 3.65, *p* = 0.004] among the brain regions examined. In the LS, more NeuN-labeled cells that were also labeled for GAD were found in rostral (*p* = 0.018) and caudal (*p* = 0.014) LSI than in LSD. At the caudal LS, less GAD-labeled cells that were also immunoreactive for NeuN were observed in LSV than in LSD (*p* = 0.005) and LSI (*p* = 0.006). Overall, the percentage of colocalization of GAD/NeuN tended to be slightly lower than NeuN/GAD. When comparing the ratio of NeuN/GAD and GAD/NeuN in each brain region of interest, one-way ANOVA revealed a higher colocalization ratio of NeuN/GAD than GAD/NeuN in rostral LSD (*p* = 0.001), caudal LSD (*p* < 0.001), Cg (*p* = 0.002) and MPOA (*p* = 0.001).

### Distribution, staining pattern, and co-localization of neurons immunoreactive for calcium-binding proteins in the LS, Cg, and MPOA

**Figure 10:**
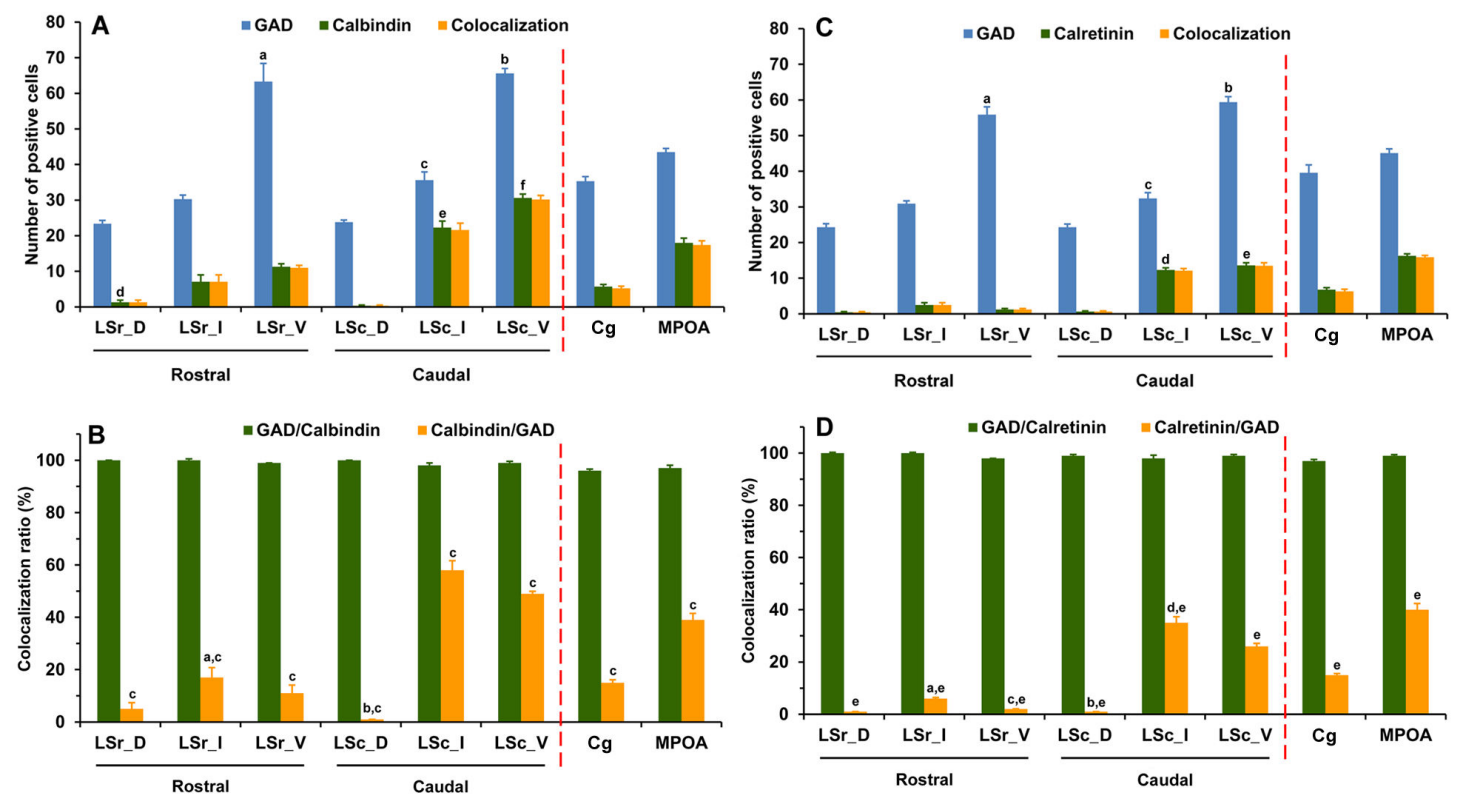
Expression and colocalization of neurons expressing mRNAs for GAD65/GAD67 (GAD) and immunoreactivity for calcium binding proteins in the LS, Cg and MPOA. Each bar represents the mean+SEM obtained from four mice. A: number of calbindin-immunoreactive cells: ^a^ *p* < 0.001 *versus* LSr_D and LSr_I, ^b^ *p* < 0.05 *versus* LSc_D and LSc_I, ^c^ *p* < 0.001 *versus* LSc_D, ^d^ *p* < 0.05 *versus* LSr_I and LSr_V, ^e^ *p* < 0.001 *versus* LSr_I and LSc_D, ^f^ *p* < 0.01 *versus* LSr_V, LSc_D and LSc_I; B: ratio of colocalization of GAD and calbindin. ^a^ *p* < 0.001 *versus* LSc_I, ^b^ *p* < 0.001 *versus* LSc_I and LSc_V, ^c^ *p* < 0.001 *versus* GAD/Calbindin; C: number of calretinin-immunoreactive cells, ^a^ *p* < 0.001 *versus* LSr_D and LSr_I, ^b^ *p* < 0.05 *versus* LSc_D and LSc_I, ^c^ *p* < 0.001 *versus* LSc_D, ^d^ *p* < 0.001 *versus* LSr_I and LSc_D, ^e^ *p* < 0.001 *versus* LSr_V and LSc_D; D, ratio of colocalization of GAD and calretinin, ^a^ *p* < 0.05 *versus* LSr_D and LSr_V, ^b^ *p* < 0.001 *versus* LSc_I and LSc_V, ^c^ *p* < 0.001 *versus* LSc_V, ^d^ *p* < 0.001 *versus* LSc_V, ^e^ *p* < 0.001 *versus* GAD/Calretinin.

**Figure 11:**
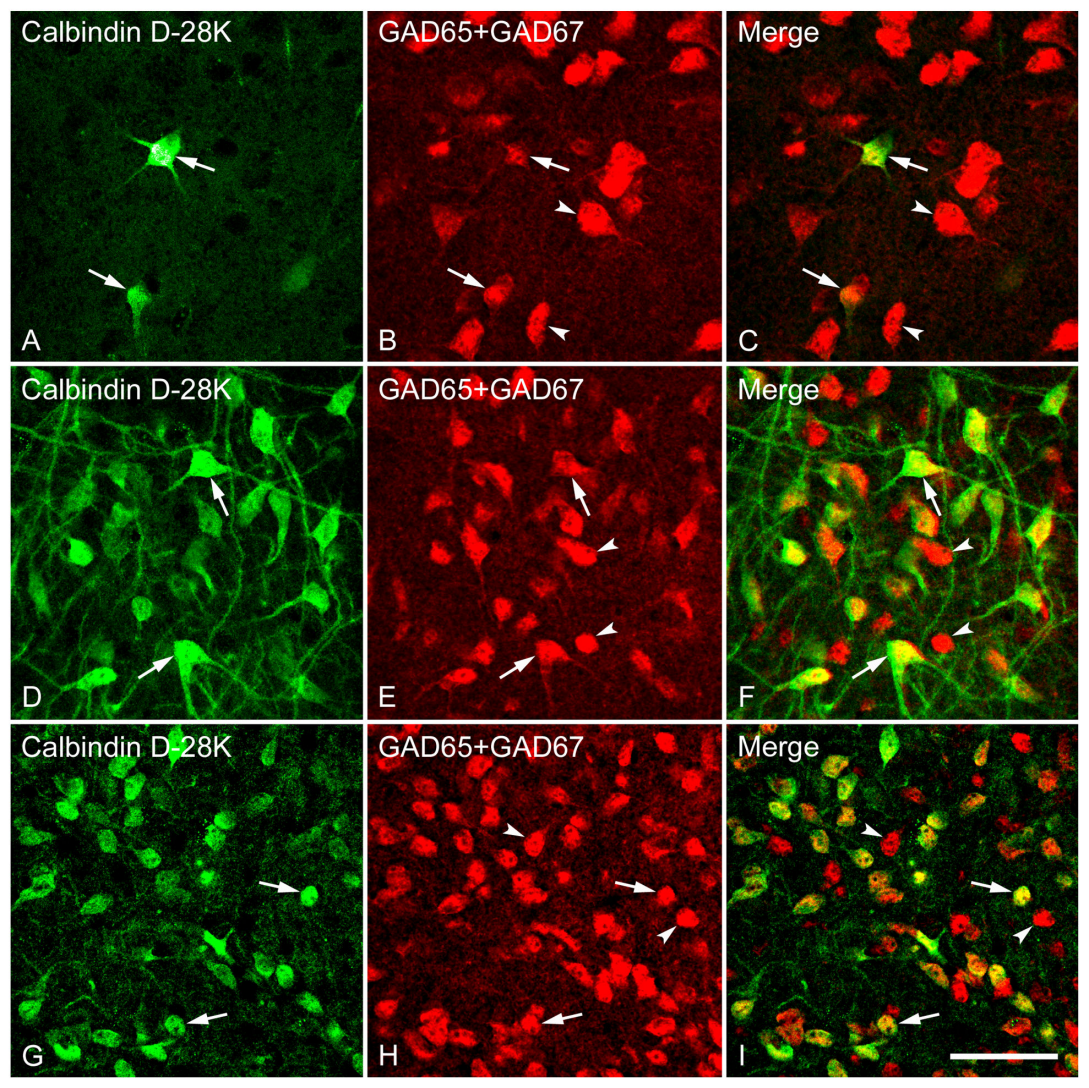
Double fluorescence in situ hybridization and immunohistochemistry labeling of neurons expressing mRNAs for GAD65/GAD67 (GAD) and calbindin-immunoreactivity in the LSD (A–C), LSI (D–F) and LSV (G–I). High magnification images show the colocalization of neurons expressing GAD and CB. Two typical examples of double-labeled (arrows) or single-labeled (arrowheads) neurons in each subdivision are indicated. Note that in all three subdivisions of the lateral septum, GAD and CB are highly coexpressed in a single cell, and most CB-immunoreactive cells express GAD mRNA (GABAergic). Scale bar = 50 µm.

**Figure 12:**
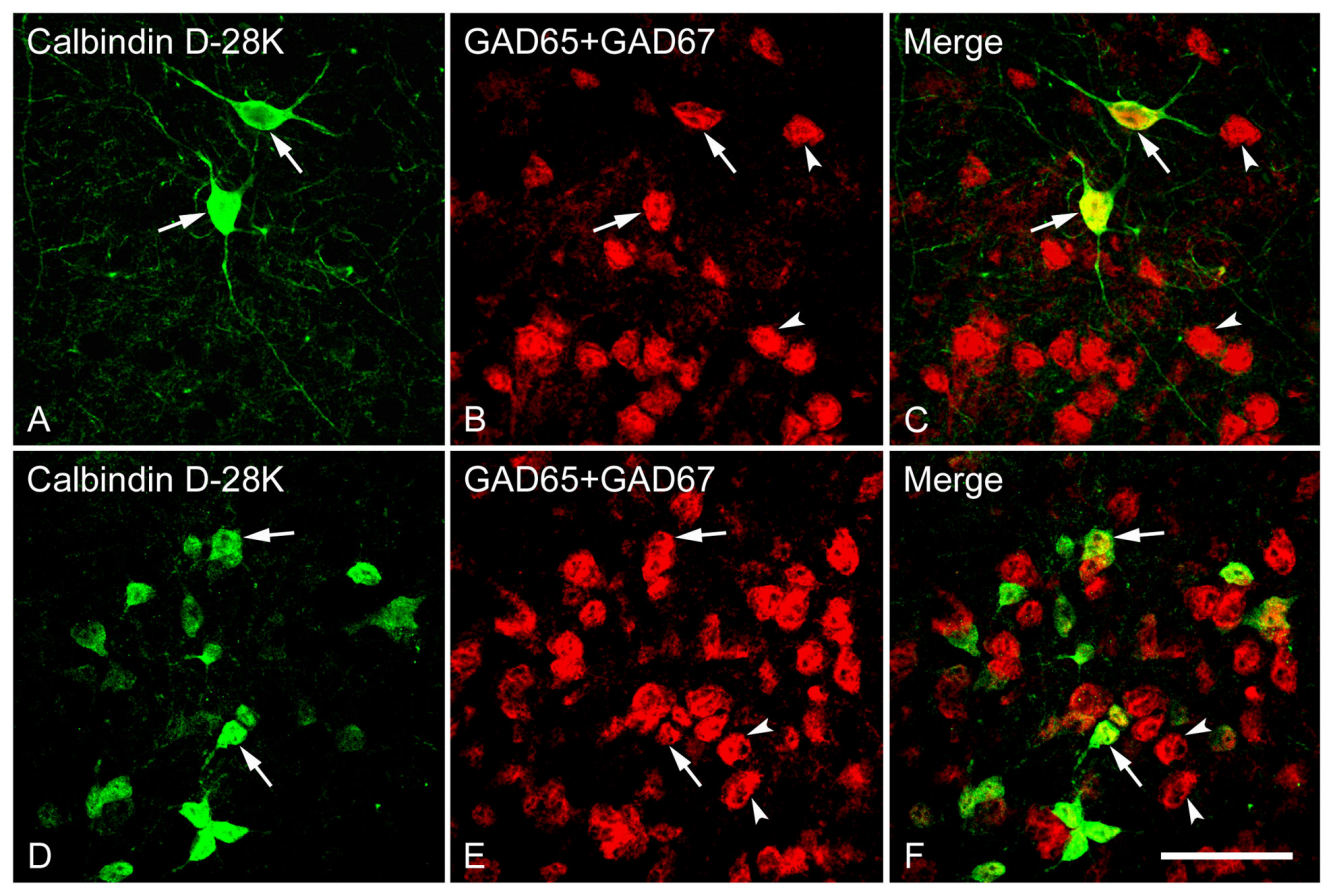
Double fluorescence in situ hybridization and immunohistochemistry labeling of neurons expressing mRNAs for GAD65/GAD67 (GAD) and calbindin-immunoreactivity in the Cg (A–C) and MPOA (D–F). High magnification images show the colocalization of neurons expressing GAD and CB. Two typical examples of double-labeled (arrows) or single-labeled (arrowheads) neurons are indicated. Note that GAD and CB are highly coexpressed in a single cell, and most CB-immunoreactive cells express GAD mRNA (GABAergic). Scale bar = 50 µm.

The general pattern of calbindin (CB) staining observed in the LS was very similar to the findings of previous studies [48–51]. CB-immunoreactive neurons were heterogeneously distributed throughout the LS. One-way ANOVA [*F*(7,24) = 82.76, *p* < 0.001] revealed a significant regional difference in the density. As illustrated in Figure 10A, at both rostral and caudal levels, CB was only rarely seen in the LSD, while a relatively higher level of expression was observed in the LSI and LSV. Further, more CB-immunoreactive neurons were preferentially located in the caudal over rostral LSI and LSV. In the caudal LSI, darkly-stained round or fusiform somata and long dendrites were observed (Figure 11D), giving the cell a bipolar appearance. The cell bodies of CB-immunoreactive neurons were scattered throughout the abundant neuropil, while in the caudal LSV the neuropil labeling with CB is virtually absent (Figure 11D, G). In the Cg (Figure 12A–C) and MPOA (Figure 12D–F), CB-immunoreactive neurons were unevenly distributed.

**Figure 13:**
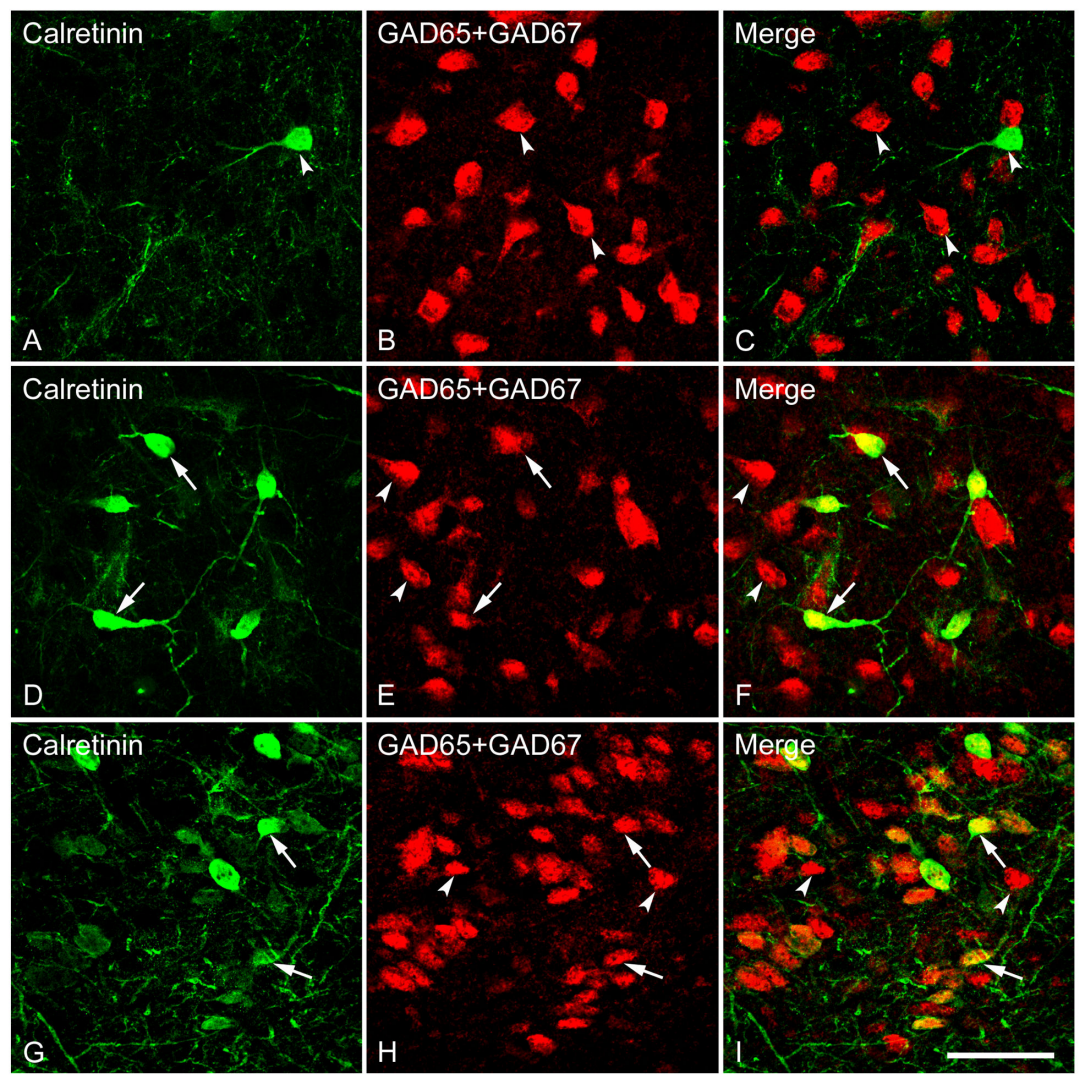
Double fluorescence in situ hybridization and immunohistochemistry labeling of neurons expressing mRNAs for GAD65/GAD67 (GAD) and calretinin-immunoreactivity in the LSD (A–C), LSI (D–F) and LSV (G–I). **High magnification images show the colocalization of neurons expressing GAD and CR**. Typical examples of double-labeled (arrows) or single-labeled (arrowheads) neurons in each subdivision are indicated. Note that in all three subdivisions of the lateral septum, GAD and CR are highly coexpressed in a single cell, and most CR-immunoreactive cells express GAD mRNA (GABAergic). Scale bar = 50 µm.

**Figure 14:**
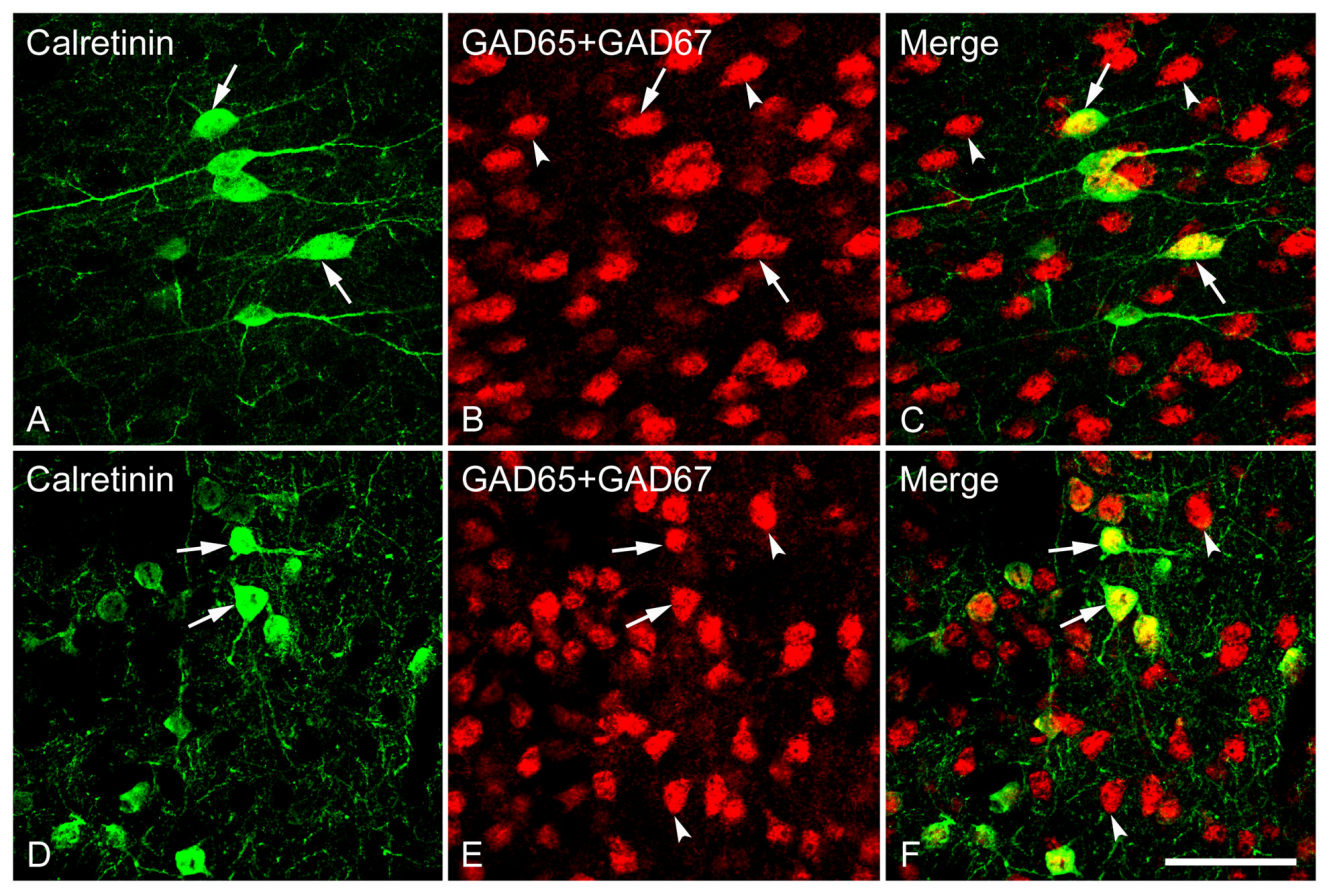
Double fluorescence in situ hybridization and immunohistochemistry labeling of neurons expressing mRNAs for GAD65/GAD67 and calretinin-immunoreactivity in the Cg (A–C) and MPOA (D–F). High magnification images show the colocalization of neurons expressing GAD and CR. Two typical examples of double-labeled (arrows) or single-labeled (arrowheads) neurons are indicated. Note that GAD and CR are highly coexpressed in a single cell, and most CR-immunoreactive cells express GAD mRNA (GABAergic). Scale bar = 50 µm.

One-way ANOVA [*F*(7,24) = 174.84, *p* < 0.001] revealed a heterogeneous distribution of calretinin (CR)-immunoreactive neurons among subdivisions of the LS, Cg and MPOA. At the caudal LS, no or rare CR-immunoreactivity was observed in LSD, while a relatively higher level of expression was detected in the LSI and LSV (Figure 10C). There was a gradual increase in the number of cells displaying CR-immunoreactivity from the rostral to the caudal LS, as evidenced by numerous cells found in caudal versus rostral LSI and LSV (Figure 10C). In the caudal LSI, intensely stained somata with long dendrites were frequently observed (Figure 13D). Notably, in the Cg the CR-immunoreactive neurons were often oriented horizontally and bipolar-like CB-containing cells with long and thin dendritic processes were interspersed among the GABAergic neurons (Figure 14A).

**Figure 15:**
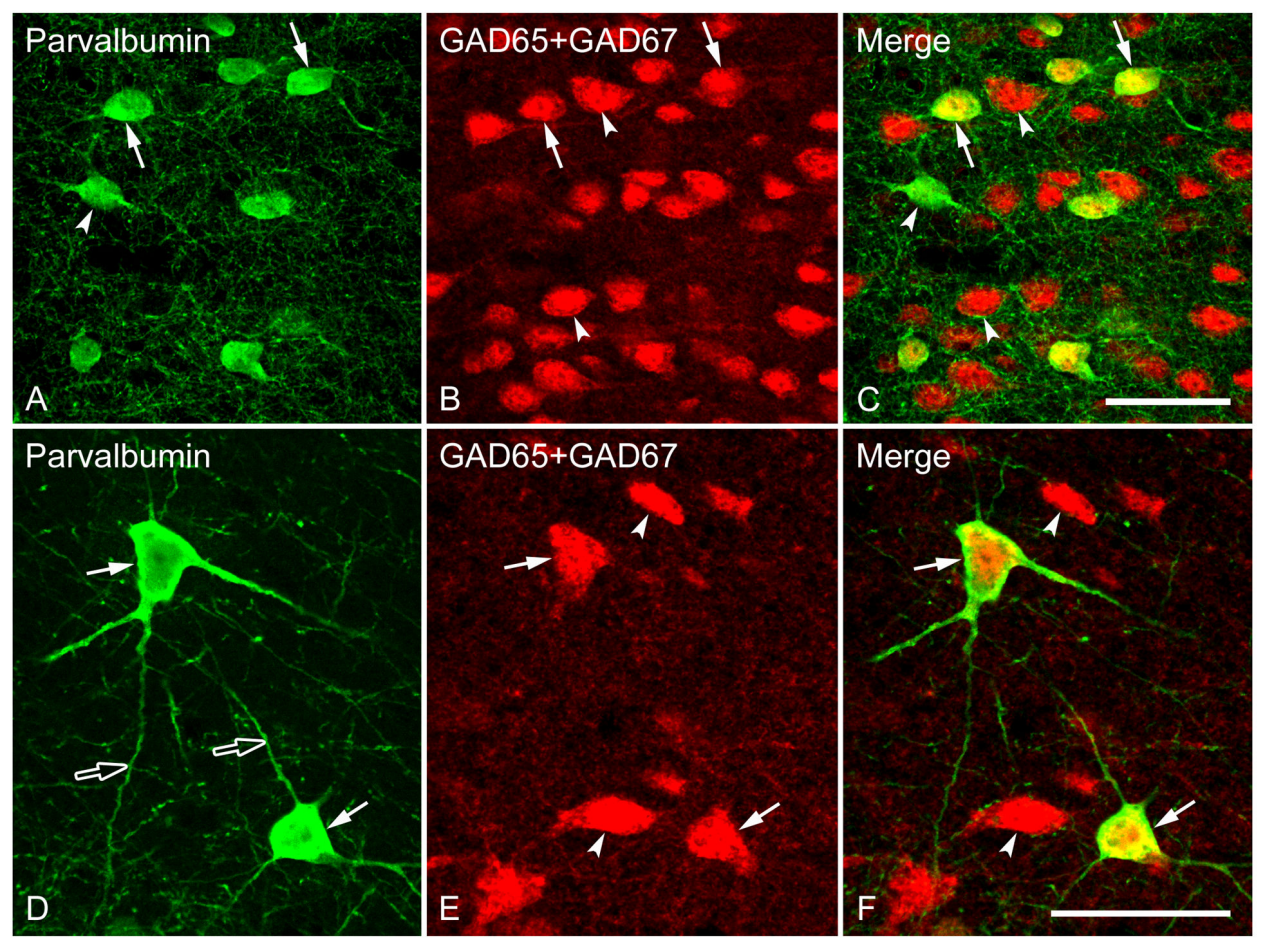
Double fluorescence in situ hybridization and immunohistochemistry labeling of neurons expressing mRNAs for GAD65/GAD67 (GAD) and parvalbumin-immunoreactivity in the Cg (A–F). High magnification images show the colocalization of neurons expressing GAD and PV. Typical examples of double-labeled (arrows) or single-labeled (arrowheads) neurons are indicated. Widely spreading dendrites (D, open arrows) and cell bodies (D, arrows) of PV-immunoreactive neurons are clearly stained. Note that GAD and PV are highly coexpressed in a single cell, and most PV-immunoreactive cells express GAD mRNA (GABAergic). Scale bars = 50 µm in A-C; 50 µm in D–F.

PV-immunoreactive neurons were scattered in the Cg (Figure 15A, D), but were not detected in LS and MPOA, consistent with previous findings of a glaring lack of detectable constitutive expression of PV in LS [52], as well as in the Allen Brain Atlas database (www.brain-map.org). In the Cg, an almost homogeneously distributed immunoreactivity for PV was found in the nuclei, cell bodies and dendrites, and PV-immunoreactive cells were intermingled with GAD mRNA-positive neurons (Figure 15C).

To examine the degree of colocalization of GABAergic neurons by GAD mRNAs and cell populations containing immunoreactivity for each of the calcium-binding proteins CB, CR and PV (markers for GABAergic interneurons) in the LS, we double-labeled LS sections using FISH identifying GABAergic neurons and immunohistochemistry recognizing calcium-binding proteins. As illustrated in Figure 10B, almost all the CB- immunoreactive neurons were also found to contain somal GAD mRNAs (98-100%) across the rostrocausal LS. In contrast, the proportion of GABAergic neurons displaying CB-immunoreactivity largely varied depending on the regions examined (1-58%). One-way ANOVA demonstrated a significant difference in colocalization ratio of Calbindin/GAD [*F*(7,24) = 73.92, *p* < 0.001], but not GAD/Calbindin among the brain regions of interest. In regard to the extent of colocalization of Calbindin/GAD, more GAD-positive cells that also contained CB-immunoreactivity were found in caudal LSI and LSV compared to corresponding rostral LSI and LSV (both *ps* < 0.001). At the caudal LS, CB-labeled GABAergic cells were preferentially located in LSI and LSV (both *ps* < 0.001) over in LSD. In general, the percentage of colocalization of GAD/Calbindin tended to be higher than Calbindin/GAD. One-way ANOVA revealed a higher colocalization ratio of Calbindin/GAD than GAD/Calbindin in all brain regions of interest (all *ps* < 0.001).

As shown in Figure 10D, almost all CR-immunoreactive neurons also expressed GAD mRNAs in all brain regions examined (98-100%), while only a small proportion of GAD mRNA-expressing neurons displayed CR-immunoreactity (1-35%), suggesting that CR-immunoreactive neurons were present in a subset of GABAergic cells. One-way ANOVA revealed a significant difference in colocalization ratio in Calretinin/GAD [*F*(7,24) = 168.28, *p* < 0.001], but not in GAD/Calretinin among the brain regions of interest. Of note, no or very rare GAD-labeled neurons in both rostral and caudal LSD were found to be CR-immunoreactive. At the rostral LS, more CR-labeled GABAergic neurons were observed in LSI than in LSD (*p* = 0.044) and LSV (*p* = 0.047). At the caudal LS, less CR-labeled GABAergic neurons were located in LSD than in LSI and LSV (both *ps* < 0.001). The number of CR-labeled GABAergic neurons was higher in the caudal LSI and LSV when comparisons were made between the rostral and caudal levels (*ps* < 0.001). In general, the percentage of colocalization for GAD/Calretinin is higher than Calretinin/GAD. One-way ANOVA revealed a higher colocalization ratio of Calretinin/GAD than GAD/Calretinin in all brain regions of interest (all *ps* < 0.001).

In the Cg, approximately 20% of GABAergic neurons expressed PV-immunoreactivity, whereas 98% of PV-immunoreactive neurons were GABAergic. Because of the lack of PV immunoreactivity in the LS and MPOA, the percentage of colocalization of the two cell types was not calculated.

## Discussion

Differing methodologically from most, if not all, previous related research, the present study quantitatively assessed the neurochemical phenotype of GABAergic neurons in the lateral septum by utilizing double fluorescent nonradioactive in situ hybridization and immunohistochemistry with TSA. We demonstrated for the first time that nonradioactive ISH with TSA is a reliable means for the identification of GABAergic neurons on the basis of unambiguously and consistently labeled cell bodies. With this specific and sensitive assay, we observed that GABAergic neurons were abundantly located within the entire LS, with the highest density in the ventral subdivision of LS. Colocalization study further revealed that the vast majority of GABAergic neurons coexpressed mRNAs for both GAD65 and GAD67 in the same cells of LS, suggesting the existence of dual enzyme systems for GABA synthesis. In addition, double labeling for GAD and neuronal phenotypic markers showed that almost all neurons in LS (>90%) are GABA-positive and that GABAergic cell populations are neurons rather than glial cells. Virtually all the neurons containing immunoreactivity for the calcium-binding proteins calbindin and calretinin, but not parvalbumin, were GABAergic, while the proportion of GABAergic neurons that also express calcium-binding proteins largely varied depending on the subdivision of interest.

### Colocalization of neurons expressing mRNAs for GAD65 and GAD67 in the LS

In the mammalian brain, GABA production is catalyzed by two isoforms of glutamic acid decarboxylase (GAD), GAD65 (GAD2) and GAD67 (GAD1) from its precursor glutamate [53–55]. Both GADs, together with GABA, have served as reliable and specific markers for GABAergic neurons. Previous immunohistochemical studies with antisera against GAD show that GAD-immunoreactive neurons (GABAergic) were unevenly expressed in the different subdivisions of LS [23,25]. Although immunoreactivity for both GADs was present in the somata, proximal dendrites and also the puncta resembling axon terminals [13,23,25,56], GAD65 immunoreactivity was found to be preferentially located in axon terminals, whereas GAD67 was preferentially distributed in the cytoplasm of neuronal somata [57]. Similar findings for subcellular localization of the two GADs were obtained when using the GABA antisera [22,58,59]. In marked contrast to the immunohistochemical methods, cell labeling by ISH assay is primarily confined to the cytoplasm of neuronal cell bodies, with little or no staining of punctate structures [35,36,57,60], in congruence with our present observations. The clear labeling of cell bodies by ISH techniques allows us to more precisely locate neurons and clarify their neuronal phenotype, in particular for GABAergic neurons. Given the differences in subcellular localization, regional expression level, biochemical characteristics, and roles in the regulation of multiple behaviors [36,53–55,60], it is necessary to determine the extent of colocalization of the two GAD isoforms in individual GABAergic cells. Consistent with previous ISH studies showing high percentage of colocalization of the two GADs in the same neurons in several brain regions [35,61], we found the overwhelming majority of GABAergic neurons in the mouse LS contain mRNAs encoding both GAD65 and GAD67, suggesting that the LS GABAergic neurons have the potential to synthesize GABA via both GADs. The presence of dual forms of GAD in the same GABAergic cell populations may indicate that GAD65 and GAD67 act in tandem to achieve their regulatory role in GABA synthesis, which contributes to the functional diversity of GABAergic neurons, although each GAD form plays a unique role in the total GABA synthesis.

### Colocalization of cells expressing GAD mRNAs with NeuN in the LS

NeuN, a neuronal nuclear protein, serves as a specific marker for the vast majority of neurons in the central and peripheral nervous systems [62,63]. Although NeuN has been extensively used in assessing neuronal loss and neurogenesis [64–66], a detailed study for characterizing the phenotype of GABAergic cell populations in the LS with NeuN has not previously been undertaken. To address this issue, we conducted a double-labeling experiment. We observed a very rich expression of NeuN-immunoreactive cells throughout the LS, with neurons more numerous in the LSV than in the LSD and LSI. Morphologically, the NeuN immunoreactivity was primarily concentrated in the nuclei, though to a lesser extent in the cytoplasm and proximal dendritic processes, quite similar to the profiles of NeuN-immunoreactivity as described in previous studies [62,63]. More importantly, using improved fluorescence ISH and immunohistochemistry with TSA, we demonstrated that in the LS, virtually almost all GABAergic cells also exhibited NeuN-immunoreactivity, suggesting that these GABAergic cells are neurons. Further, most neurons (>90%) were GABA-positive. These findings are important because they provide insight into the fundamental nature of phenotype of LS GABAergic cells.

### Colocalization of GABAergic neurons with calcium-binding proteins in the LS

Interneurons are classically identified by their expression of calcium-binding proteins (e.g., CB, CR and PV) [67,68]. The calcium-binding proteins are useful markers for identifying specific phenotypes of interneurons in the brain. GABAergic neurons have been extensively characterized using calcium-binding proteins markers in the cortex [19–21,69–72], hippocampus [11,18,73], and amygdala [24,74]. However, little quantitative information is available regarding the identities of GABAergic neurons specified by these interneuron markers in the LS. To address this issue, this study comprehensively examined the localization of calcium-binding proteins in the LS GABAergic neurons.

The distribution pattern of CB-immunoreactive neurons within the LS is in general agreement with earlier observations [48–50,75]. We showed that CB-immunoreactive neurons were only rarely seen in the LSD, while a relatively higher level of expression was observed in the LSI and LSV. The uneven expression of CB-containing cells within three subdivisions of the LS was further supported by the finding that CB-immunoreactive cells were preferentially located in the caudal over rostral LSI and LSV. Given the significant differences in the chemoarchitecture and neuronal connectivity among three subdivisions as well as between rostral and caudal LS [43,76–78], it can be assumed that the heterogeneous distribution of CB may be involved in the different roles that LS mediates. One important finding of the present study is that almost all the CB-immunoreactive neurons in the LS are GABAergic, quite similar to the results obtained in cortex [19,70,79] and hippocampus [11,18,73,80]. In contrast, the proportion of GABAergic cells that also expressed CB varies depending on the subdivisions and rostrocaudal levels examined, with the highest in caudal LSI (approximately 58%) and lowest in caudal LSD (less than 1%).

The presence of CR-immunoreactive neurons in the LS has been reported [51,81]. However, very few quantitative and comparative studies have been made with regard to the distribution of CR and its colocalization with GABA within the LS. CR-immunoreactive neurons were located mainly in the caudal LSI and LSV, with no or rare cells in LSD, suggestive of a heterogeneous distribution. Colocalization study showed that almost all CR-immunoreactive neurons were identified to be GABAergic throughout the entire LS (98-100%), while only a small proportion of GABAergic neurons displayed CR-immunoreactity (1-35%), suggesting that CR-immunoreactive neurons are localized in a subset of GABAergic cells. At the rostral LS, more GABAergic neurons that were also CR-immunoreactive were observed in LSI (6%) than in LSD (1%) and LSV (2%). At the caudal LS, CR-immunoreactive neurons constituted approximately 1%, 35%, and 26% of the total GABAergic cell population in LSD, LSI and LSV, respectively, indicating a higher proportion of CR-immunoreactive GABAergic neurons in the LSI and LSV than LSD. When direct comparisons of these two proteins were made, close similarities were uncovered in the distribution pattern and localization in GABAergic neurons, although it appears that the proportion of CB-immunoreactive GABAergic neurons is, in general, higher than that of CR-immunoreactive GABAergic cells due to the relatively higher expression of CB. For example, both proteins were preferentially located at the caudal level of LS over the rostral level, and were more highly expressed in LSI and LSV than LSD within the subdivisions. It is also necessary to consider that the similar distribution and colocalization with GABA are not the results of a cross-reaction between the two proteins due to the close molecular weights and highly conserved primary structure [82,83]. The specificity of CB and CR antibodies used in this study has been widely tested by Western blotting and preadsorption experiments [84]. All these experiments, together with our present study, clearly demonstrated that CB and CR antibodies used here are specific, with no cross-reaction occurring between the two antibodies, thus, precluding the possibility of cross-reaction.

Under our detection methods used here, no unequivocal immunoreactivity for PV was detected in the LS. This raises questions of whether the absence of PV immunoreactivity truly reflects the lack of PV expression or simply indicates a level of expression that is still too low to be detected. It appears that the lack of detectable, constitutive PV in the LS reflects an actual lack of expression rather than an undetectable level of expression, as previous studies using both immunohistochemistry and ISH were not able to detect a clear PV expression [52], and in the Allen Brain Atlas database (www.brain-map.org).

Although the specific roles of each of calcium-binding proteins are not fully understood, calcium-binding proteins may serve as useful markers for the classification of GABAergic neurons in the LS. On the basis of expression of different calcium-binding proteins in distinct subpopulations, LS GABAergic neurons can be classified into at least two subtypes: 1) neurons containing only CB immunoreactivity; 2) neurons displaying only CR immunoreactivity. Since we did not examine the colocalization of CB and CR in this study, the existence of a third subpopulation of GABAergic neurons immunoreactive for both CB and CR could not be excluded. As a significant proportion of GABAergic neurons, particularly in the rostral LS, did not express the defined calcium-binding proteins CB, CR and PV, a largely unanswered question is what the phenotype of the remaining GABAergic cell populations is. It is posited that they may contain other interneuron markers such as neuropeptides as observed in cortex [85,86], or be projection neurons. This interpretation is further supported by the concept that LS is predominantly composed of GABAergic projection neurons, and expresses distinct pattern of neuropeptides within each rostrocaudal subdivision [42,43,77]. LS contains a large number of projection neurons and has extensive reciprocal or non-reciprocal connections with multiple neuronal sites, including medial prefrontal cortex, hippocampus, hypothalamus, ventral tegmental area, raphe nucleus and amygdala [42,43,77]. The findings here suggest that most of the projection neurons contain GABA, but what other neurotransmitters are contained in these projections still needs to be determined in detail. Although the GABAergic neurons that express distinct interneuron markers of calcium-binding proteins have been suggested to be associated with neuronal connectivity and physiological activities in the cortex and hippocampus [87–89], to date, very little is known about the roles of the GABAergic interneurons in the LS. As LS GABAergic neurons display unique pattern of colocalizations with each of calcium-binding proteins, it is likely that each subtype of interneurons identified plays distinct physiological functions and constructs different neuronal inhibitory circuitries, which may ultimately contribute to their associated behavioral regulation.

### Characterization of GABAergic Neurons in the Cg and MPOA and their relevance to previous observations

Like LS, cell populations in the Cg and MPOA displayed quite similar patterns of colocalization of GAD65 and GAD67 as well as GAD and NeuN, indicating that the vast majority of neurons are GABAergic and contain dual enzyme systems in single cells within these two brain regions. In addition, neurons in the Cg and MPOA expressed a phenotype similar to the LS as identified by calcium-binding proteins CB and CR, but not PV. These findings are supported by previous observations showing that neurons in the cortex and MPOA are largely GABAergic and contain calcium-binding proteins CB, CR and/or PV [19,20,70,90–92], thus validating the efficacy of our approaches and expanding results to additional regions.

### Technical considerations

The difficulties in unambiguously identifying GABAergic neurons using immunohistochemical methods, as mentioned in the Introduction, have long made the precise quantitative analysis of GABAergic neurons problematic. To overcome the limitations of immunohistochemical assay, conventional nonradioactive/radioactive ISH study using sections labeled for GAD65 and GAD67 mRNAs was undertaken [35,36,57,61]. Despite the high sensitivity of visualization, radioactive ISH methods did not allow unambiguous identification of the cell bodies of the majority of GABAergic neurons due to scattering of the emitted radiation and associated silver grains beyond the boundaries of the cell bodies, making a precise assessment difficult [60,61]. In contrast to radioactive ISH, nonradioactive ISH is less sensitive, restricting their usefulness for detection of relatively high abundance mRNAs or a single probe [93,94].

The novel aspect of this study is the use of fluorescent nonradioactive ISH with TSA for identification of GABAergic neurons, which was not reported in previous observations. Tyramide signal amplification (TSA), an enzyme mediated detection method that utilizes the catalytic activity of horseradish peroxidase (HRP) to generate highly amplified signals of a target protein or mRNA *in situ*, is ideal for detection of low abundance protein and mRNA [40,95]. Aside from the effect of dramatically enhancing intensity of signals (up to 100-fold), another advantage of TSA method is better single-cell resolution because the amplified signals are deposited at or proximal to the HRP enzyme site without diffusion [37]. The TSA plus fluorescence systems technology uses HRP to catalyze the deposition of a fluorophore-labeled tyramide amplification reagent onto tissue sections, resulting in dramatically increased fluorescent signals, which then can be detected by fluorescence visualization techniques. Fluorescence ISH and immunohistochemical methods with TSA have been demonstrated to provide considerable enhancement of detection sensitivity compared to conventional fluorescence ISH and immunofluorescence applications [40,41,96–98]. Further, double fluorescent ISH and/or immunohistochemical methods with TSA have been successfully used for detection of colocalization of two different mRNAs or mRNA and protein in a single cell [99]. The high level of cellular resolution and intensely stained cell bodies in single neuron labeled for each GAD mRNA that were obtained in this study enabled us to precisely and reliably determine GABAergic neurons.

## Conclusions

Overall, the nonradioactive ISH with TSA method presented in this study has been demonstrated to be a sensitive and reliable means of detecting GABAergic neurons and clarifying their neuronal phenotypes when used in concert with immunohistochemical assays. Despite the potential of an overwhelming majority of neurons in the LS for synthesizing GABA via the dual enzyme systems GAD65 and GAD67, it still remains to be determined how the two GADs work synergistically to achieve their catalytic effect. It is noteworthy that the finding that GABAergic neurons within each of subdivision of the LS display unique expression of calcium-binding proteins CB and CR represents an important step towards delineating the structure, neuronal connectivity and functional significance of the LS. Thus, further work is required to address how GABAergic calcium-binding protein-containing neurons are involved in the construction of inhibitory neural networks of LS, and consequently contribute to various LS-mediated behavioral processes.

## Material and Methods

### Animal subjects

Naïve, virgin female mice (2.5 months old, 25-35 g) from outbred hsd:ICR strain ( *Mus* *domesticus* ) (Harlan, Madison, WI) were used in this study. Virgin female mice were selected because they provide a fundamental basis of the distribution, morphology and chemical phenotype of neurons. We recently found that GAD expression is elevated in maternal mice [100], so the virgin mice provide an important conservative baseline for GAD expression. Female mice were maintained in groups with 2-3 mice per cage in our colony for at least 2 weeks prior to the start of the study. All animals were housed in the same room on a 14:10 light/dark cycle with lights on at 06:00 h CST. Female mice were given ad lib access to regular mouse chow (Harlan) and tap water. All experimental procedures were performed in compliance with the guidelines of the National Institutes of Health Guide for the Care and Use of Laboratory Animals and were approved by the Animal Care and Use Committee of the University of Wisconsin. All efforts were made to minimize the number of animals used and their suffering.

### Staging of virgin females

Immediately prior to perfusion, virgin females were examined for stage of estrous cycle using a vaginal lavage [101,102]. Female mice in only vaginal diestrus were perfused and included in this study.

### Brain slice preparation

On the day of perfusion between 10:00 and 12:00 h, virgin mice were lightly anaesthetized with isoflurane, further deeply anesthetized with 0.15 ml of sodium pentobarbital, and then transcardially perfused with ~50 ml ice-cold saline, followed by 4% paraformaldehyde in 0.1 M phosphate buffer (PB; pH 7.4). Brains were removed and postfixed overnight in the same fixative and then cryoprotected in 30% sucrose in cold 0.1 M PB for two days. Brains were snap frozen on a platform and 30 micron thick coronal sections were sliced on a cryostat (Leica, CM1850, Bannockburn, IL, USA) and stored in cryoprotectant solution [103] at -20 °C until processing. The lateral septum (LS) sections including the dorsal (LSD), intermediate (LSI) and ventral (LSV) subdivisions were collected from Bregma 1.045 to 0.02 mm (Figure 1). As controls, cingulate cortex (Cg) and medial preoptic area (MPOA) were also included (Figure 1). Each mouse provided one sample of each subdivision of LS.

### Probe design

We used a cocktail of oligonucleotide probes directed against different parts of each GAD mRNA to enhance sensitivity of detection [104]. To generate highly specific probes and ensure that the sequence chosen for the probes does not cross-hybridize with each other, we designed probes for GAD65 mRNAs that hybridize to its complementary RNA of encoding sequence and probes for GAD67 mRNAs that hybridize to its complementary RNA of non-coding sequence near the 3’ terminal of the RNA (Figure 2). In addition, there was no overlap of sequences among all the probes and the probes designed for one GAD had no homology to any portion of the other GAD (Figure 2). Such probe designs allowed us to eliminate the cross-hybridization between the two probes and maximize the sensitivity of detection. Two sets of probes for GAD65 mRNA that had identical sequences were labeled with either biotin (for double FISH labeling) or digoxigenin (DIG) (for double FISH and IHC), while probes for GAD67 mRNA were labeled with digoxigenin. Combinations of labels were summarized in Table 1. Biotin or DIG-labeled oligonucleotide probes used in this study were synthesized by Integrated DNA Technologies, Inc., Coralville, Iowa, USA and probe sequences were detailed in Table 2 and Figure 2. The specificity of each probe has been verified through sequence identity search in NIH GenBank databases.

**Table 1:** Combinations of the labels that were used in the fluorescence double-labeling study.

	Probe	Label	Primary antibody	Visualization
Double FISH	GAD65	Biotin		Alexa Fluor 488-conjugated tyramide (green)
	GAD67	Digoxigenin		Cy3-conjugated tyramide (red)
Double FISH and IHC	GAD65 & GAD67	Digoxigenin		Cy3-conjugated tyramide (red)
			NeuN	Alexa Fluor 488-conjugated donkey anti-mouse antiserum (green)
			Calbindin D-28K	Alexa Fluor 488-conjugated donkey anti-mouse antiserum (green)
			Calretinin	Alexa Fluor 488-conjugated donkey anti-mouse antiserum (green)
			Parvalbumin	Alexa Fluor 488-conjugated donkey anti-mouse antiserum (green)

**Table 2:** Oligonucleotides used for GAD probes.

Probe		Sequence (5’ to 3’)
GAD65	Probe 1	TTCCTGTATCTTTCTGAGTACCAAAATGCCCATCCATATGGAGTGGGAGAATGCTTCCAG
	Probe 2	ACAGCAAGGGCCCAAAGCTACAGTCATACTGAGGATCAGTATGTCTAGTCAAATAAGGTC
	Probe 3	CGAGCAAGGCTCCTAGAAATTTTGAGGAAGAATCTGGAGAAGAAGGGGATTGGAGATCCA
GAD67	Probe 1	CCATCTCAAACTCTTCTCTGTTTTTAATCTTGGCGTAGAGGTAATCAGCCAGCTCCAGGC
	Probe 2	CCTGACCCAACCTCTCTATCTCCTCAATGAGGAAATCGATGTCAGACTGGGTGGCGGCTG
	Probe 3	TAATTTCCTTCAGTGAGATGGCCTAGATGTGTCAGCTACTGACAGAGCTGTGCTCTAGGG

### Double fluorescence in situ hybridization with TSA

Double fluorescent labeling was carried out at room temperature unless otherwise indicated. Brain sections were washed 5x 5 min in DEPC-PBS to clear cryoprotectant, and then incubated in 1.5% H_2_O_2_ in DEPC-PBS for 30 min to inhibit endogenous peroxidase activity. They were placed in 0.2 N HCl for 20 min, and acetylated in 0.25% acetic anhydride in 0.1 M triethanolamine-HCl (pH 8.0) for 10 min. Sections were pre-hybridized for 2 h at 37° C in hybridization buffer containing 5x SSC, 50 µg/ml heparin, 0.001 M EDTA (pH 8.0), 50% formamide, 1x Denhardt’s solution, 0.1% tween-20, 0.25 mg/ml Yeast tRNA, 10% Dextran sulphate. Following pre-hybridization, they were hybridized by incubating in hybridization buffer containing a cocktail of probes for GAD65 (biotin-labeled) and GAD67 (DIG-labeled) (1.0 µg/mL) at 37° C overnight. Post-hybridization washes were performed sequentially 2x 30 min at 37° C in 50% formamide/2x SSC, 1x15 min in wash buffer containing 0.05% Tween-20 in TBS (0.15 M NaCl in 0.1 M Tris-HCl, pH 7.5), 1x 30 min in 50% formamide/2x SSC, 1x 30 min in 50% formamide/0.2x SSC, and 1x 15 min in wash buffer. They were incubated for 1 h in blocking buffer (2% blocking reagent from Roche Applied Science in TBS), followed by incubating for 1 h with streptavidin (SA)-HRP diluted 1:100 in blocking buffer. After washing 3x 10 min in wash buffer, sections were subsequently incubated for 30 min in Alexa Fluor 488-conjugated tyramide (Molecular Probes, Eugene, OR, USA) by diluting TSA stock solution 1:100 in 0.0015% H_2_O_2_/amplification buffer. They were washed 3x 5 min with wash buffer and incubated for 30 min in 3% H_2_O_2_ in TBS to quench residual peroxidase activity from the initial TSA reaction. They were incubated for 60 min with Anti-Digoxigenin-POD, Fab fragments (Roche Diagnostics GmbH, Mannheim, Germany) diluted 1:100 in blocking buffer, washed 3x 10 min with wash buffer, then incubated for 10 min in Cy3-conjugated Tyramide (TSA^TM^ Plus Cyanine 3 kit, PerkinElmer, Waltham, MA, USA) by diluting TSA stock solution 1:50 in 1x Amplification Diluent. Following washing 3x 10 min with wash buffer, sections were mounted onto slides using DePeX mounting medium (Serva, Heidelberg, Germany) and stored in the dark room at 4° C. As controls, hybridization with sense probe, quenching of HRP activity prior to tyramide–coupled fluorochromes, or omission of the Anti-Digoxigenin-POD or the SA-HRP abolished all ISH signal. The three different probes for each GAD mRNA gave the same pattern of labeling.

### Double fluorescence in situ hybridization and immunohistochemistry with TSA

The procedures prior to hybridization were identical to those of double FISH with TSA as detailed above. Following pre-hybridization, sections were incubated in hybridization buffer containing a mixture of both GAD65 and GAD67 probes that were labeled with digoxigenin (~1.0 µg/mL) at 37° C overnight. Sections were sequentially rinsed 2x 30 min at 37° C in 50% formamide/2x SSC, 1x 15 min in wash buffer, 1x 30 min in 50% formamide/2x SSC, 1x 30 min in 50% formamide/0.2x SSC, and 1x 15 min in wash buffer. They were incubated for 1 h in blocking buffer, then incubated for 1 h with Anti-DIG-HRP diluted 1:100 in blocking buffer, washed 3x 10 min with wash buffer, then incubated for 10 min in Cy3-conjugated tyramide (TSA^TM^ Plus Cyanine 3 kit) by diluting TSA stock solution 1:50 in 1x Amplification Diluent. After washing 3x 10 min with wash buffer, sections were incubated for 30 min in 3% H_2_O_2_ in TBS to quench peroxidase activity from the initial TSA reaction. After washing and blocking, sections were incubated with the following primary antibodies in blocking buffer: mouse anti-NeuN (Millipore, MAB377, Bilerica, MA, USA; diluted 1:100), mouse anti-calbindin D-28K (Swant, 300, Bellinzona, Switzerland, diluted 1:1000), mouse anti-calretinin (Swant, 6 B3, Bellinzona, Switzerland; diluted 1:500) and mouse anti-parvalbumin (Swant, PV235, Bellinzona, Switzerland; diluted 1:100). Sections were washed 3x 10 min with wash buffer and then incubated for 2 h in Alexa Fluor 488-conjugated donkey anti-mouse antiserum diluted 1:100 in TBS. Following washing 3x 10 min with wash buffer, sections were mounted onto slides using DePeX mounting medium, air-dried and stored in the dark room at 4° C. The specificity of antibodies used in this study has previously been extensively characterized [48,84,105–107]. As controls, hybridization with sense probe, quenching of HRP activity prior to Cy3-conjugated tyramide incubation, or omission of the Anti-Digoxigenin-POD or primary antibodies completely abolished ISH signals and/or immunoreactivity.

### Quantitation of double fluorescent labeling

All confocal fluorescent images were captured sequentially using a laser scanning confocal microscope (MRC-1024; Bio-Rad, Hercules, CA, USA) connected to an inverted microscope (Zeiss, Germany) with Bio-Rad LaserSharp 2000 acquisition software. All images in each indicated sample area (Figure 1) were acquired with a screen resolution of 1024×1024 pixels using a 10x objective or a 63x objective oil immersion lens. Confocal images presented here were projected using a maximum intensity projection of z-series stacks taken at 0.5 micron step to cover the entire thickness of the lateral septum section (typically 8-10 tissue sections representing the rostral-caudal extent of the LS). For quantitative analysis of colocalization, cell counting was carried out using 63x magnification photomicrographs in three subdivisions (LSD, LSI and LSV) throughout the rostrocaudal extent of the LS, Cg and MPOA (Bregma levels approximately from 1.045 to 0.02 mm; Figure 1). The number of single-labeled cells identified by clearly stained somata was counted unilaterally in every third section within a 163 µm X 163 µm unit area. Simultaneously, double labeled cells were also numerated based on the coincidence of green and red labeling in cell bodies (yellow) showing colocalized mRNAs (GAD65 and GAD67) or mRNA and immunoreactivity (GAD and specific cell markers). The counting was performed manually by investigators blind to the labeling conditions, such as the types of probe and antibody. All confocal images were transferred to Adobe Photoshop 6.0 (Adobe Systems, San Jose, CA, USA) and then merged, with adjustments of brightness and contrast. Total data were collected from six animals for double labeling of GAD65 and GAD67 (N = 6), six animals for GADs and NeuN (N = 6), four animals for each double labeling of GADs and calcium-binding proteins (N = 4 for CB, CR, PV, respectively).

### Statistical analysis

Statistical analyses were performed using SPSS 20.0 software (SPSS Inc., Chicago, IL, USA). Data for cell number and colocalization ratio were expressed as mean ± SEM and analyzed using a one-way analysis of variance (ANOVA) followed by Tukey tests post hoc comparisons. A conventional two-tailed level of significance at the 0.05 level was required. Colocalization ratio of GAD67/GAD65 was calculated by dividing the number of neurons expressing both GAD65 and GAD67 mRNAs (colocalization) by total number of neurons expressing GAD65 mRNA, and multiplying by 100. This formula applied to the calculation of all colocalization ratios in this study. As the present study was focused on the characterization of phenotype of GABAergic neurons in the LS, no direct comparisons of the cell number and colocalization ratio were made between the LS and Cg, LS and MPOA.
